# Identifying past-year self-reported suicidality in outpatients with somatic symptom disorder using an interpretable machine-learning model: a multicenter study with an online calculator

**DOI:** 10.1186/s12888-026-07901-9

**Published:** 2026-02-18

**Authors:** Xing Wang, Shuixiu Lai, Peng Wang, Yibo Li, Yunhui Zhong, Tieshi Zhu

**Affiliations:** 1 Department of Neurosis and Psychosomatic Diseases, Huzhou Third Municipal Hospital, The Affiliated Hospital of Huzhou University, Huzhou, Zhejiang China; 2 Jiangxi Environmental Engineering Vocational College, Ganzhou, Jiangxi China; 3 Department of Language, Literature and Communication, Faculty of Social Sciences and Humanities, Vrije Universiteit Amsterdam, Amsterdam, Netherlands; 4 Department of Psychology, Education, and Child Studies, Erasmus School of Social and Behavioural Sciences, Erasmus University Rotterdam, Rotterdam, Netherlands; 5 Department of Clinical Psychology, Tianjin Medical University General Hospital, Tianjin, China; 6 The Third People’s Hospital of Ganzhou, No. 10 Zhangjiang North Avenue, Zhanggong District, Ganzhou City, Jiangxi Province China; 7 Department of Neurology, Zhanjiang Central Hospital, Guangdong Medical University, No. 236 Yuanzhu Road, Chikan District, Zhanjiang City, Guangdong Province China

**Keywords:** Somatic symptom disorder, Suicidality, Machine learning, Risk prediction, Web-based calculator

## Abstract

**Background:**

Somatic symptom disorder (SSD) is associated with an elevated risk of suicidality. However, clinically implementable tools to identify outpatients with SSD who may warrant prioritized suicidality assessment remain limited. We therefore aimed to develop an interpretable model using routinely available outpatient data to stratify the likelihood of past-year self-reported suicidality.

**Methods:**

We analyzed a multicenter cross-sectional registry from 3 hospitals in Ganzhou including adults aged 18–60 years with DSM-5–defined SSD. Past-year self-reported suicidality was assessed using a prespecified binary (yes/no) item. Data were split 70/30 into training/test sets. Candidate predictors were selected by the intersection of least absolute shrinkage and selection operator and Boruta. Eight algorithms were trained with repeated 5-fold cross-validation and compared primarily by area under the receiver operating characteristic curve (AUC) and Brier score; the top model underwent calibration and decision-curve analysis. Shapley additive explanations (SHAP) provided model explanations; a Shiny web calculator was implemented.

**Results:**

Of 899 participants (median age, 33 years; 64.4% female), 19.9% reported past-year suicidality. All models showed high discrimination in the test set (AUCs > 0.900). The random forest (RANGER implementation) performed best (AUC, 0.978; 95% CI, 0.955–1.000; area under the precision–recall curve, 0.960; Brier, 0.028; accuracy, 0.967; sensitivity, 0.927; specificity, 0.977), with good calibration and favorable net clinical benefit on DCA. SHAP ranked insomnia severity index as the leading contributor, followed by the five facet mindfulness questionnaire and the repeatable battery for the assessment of neuropsychological status.

**Conclusions:**

In SSD outpatients, an interpretable RANGER-based model showed strong internal performance for classifying participants who reported past-year self-reported suicidality, and yielded favorable clinical net benefit across relevant decision thresholds. A web-based calculator illustrates potential usability in outpatient settings; external validation and prospective implementation studies are warranted before routine adoption.

**Supplementary Information:**

The online version contains supplementary material available at 10.1186/s12888-026-07901-9.

## Graphical Abstract

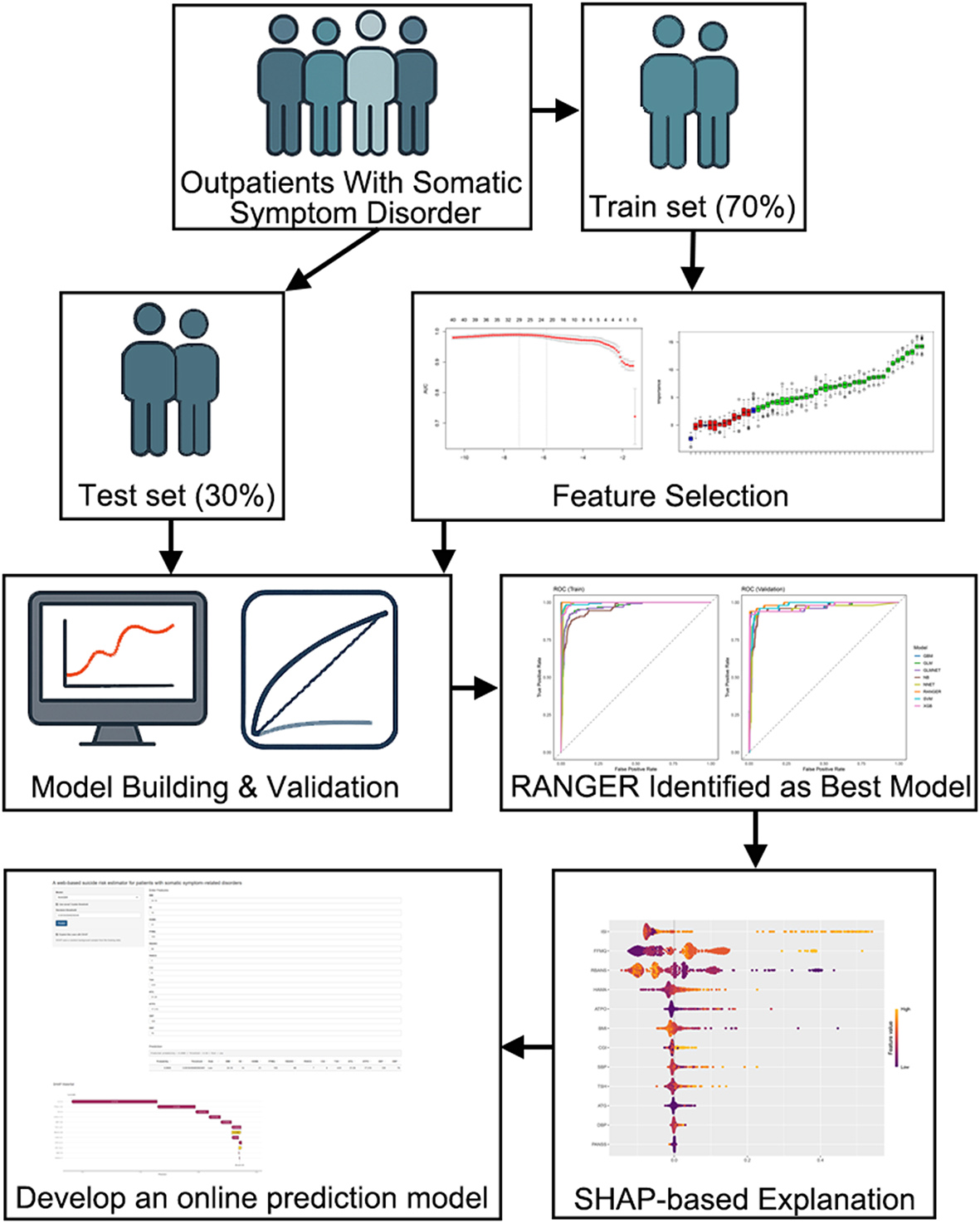

## Background

Somatic symptom disorder (SSD) is a common and functionally impairing condition characterized by persistent, distressing somatic symptoms accompanied by disproportionate health-related thoughts, feelings, or behaviors, irrespective of whether a medical explanation is identified [1–3]. Individuals with SSD experience substantial symptom-related distress and disability and often receive care across multiple clinical services, contributing to high health care utilization and costs [4–6]. SSD is also associated with increased risk of suicidal ideation, suicide attempts, and death by suicide [7, 8].

Given the substantial morbidity and mortality associated with suicidal behavior, timely identification of individuals at high risk is essential [9, 10]. In SSD, clinical attention often centers on bodily complaints, and patients frequently present to nonpsychiatric services [11, 12]. As a result, potential suicide risk may be obscured by complex somatic symptoms and overlooked in routine care [13, 14]. Prior studies have identified some associated factors—such as greater somatic symptom burden and poor sleep quality—but these individual risk factors have limited predictive value for suicidal behaviors [15, 16]. Therefore, a standardized, objective, and clinically implementable tool is needed to integrate multidomain information to assist clinicians in identifying high-risk individuals within SSD care pathways.

In recent years, machine learning (ML) has shown substantial promise for predicting mental health risk, owing to its capacity for pattern recognition and high-dimensional data processing [17, 18]. Compared with traditional statistical approaches, ML algorithms excel in learning nonlinear and interaction effects from complex clinical data, thereby improving risk stratification [19, 20]. ML has been applied to suicide-risk models in populations with depression and schizophrenia [21, 22], among others [23, 24]; however, evidence specific to outpatients with SSD—a distinct, high-risk group—remains limited [25]. This gap is likely driven by the clinical pathway of SSD: patients often present with predominant somatic complaints in outpatient medical settings, and care frequently prioritizes medical workups to exclude organic disease, contributing to high healthcare utilization [26–28]. Suicide-risk assessment may therefore be less systematic and receives limited attention in some existing management guidance for somatic symptom and related disorders [14]. In addition, SSD is clinically heterogeneous and frequently comorbid with affective and anxiety symptoms, and the lack of standardized, consistently documented outcomes can hinder model development and external validation [2, 3, 29]. Moreover, many ML models have limited interpretability and face barriers to clinical implementation, even when supplemented with Shapley additive explanations (SHAP). Web-based calculators built with Shiny may facilitate bedside use and dissemination [30, 31]. Therefore, predicting suicide risk is a critical clinical priority in patients with SSD. Accordingly, using outpatient data from three hospitals in Ganzhou, we developed interpretable machine-learning models to stratify the likelihood of past-year self-reported suicidality at the index visit. This retrospective stratification is intended as a pragmatic screening/triage approach to help identify patients who may warrant prioritized and structured suicide risk assessment. We also implemented a web-based Shiny calculator to facilitate clinical use.

## Methods

### Participants

This cross-sectional registry enrolled participants at the First Affiliated Hospital of Gannan Medical University, the Third People’s Hospital of Ganzhou, and Ganzhou People’s Hospital (Jiangxi, China) from January 2023 through April 2024. Eligible participants were Han Chinese adults aged 18 to 60 years with DSM-5 SSD and capacity to consent. Diagnoses were verified by a senior psychiatrist, with disagreements resolved during weekly case conferences. The present report addresses a research question distinct from prior analyses of this registry. Of 1068 individuals screened, 997 met inclusion criteria; 98 were excluded (pregnancy/lactation, *n* = 23; substance use disorder, *n* = 25; severe personality disorder, *n* = 13; serious physical illness, *n* = 12; declined participation, *n* = 20; other, *n* = 5), yielding a final analytic cohort of 899.

### Ethical approval

The protocol was approved by the institutional review board of the Third People’s Hospital of Ganzhou (IRB: Ethics Committee of the Institute of Third People’s Hospital of Ganzhou, Ethical code: gzsyy2024044). Written informed consent was obtained from all participants, and confidentiality was maintained. All procedures adhered to the principles of the Declaration of Helsinki.

### Outcome

The primary outcome was past-year self-reported suicidality, assessed at the index outpatient visit using a single binary (yes/no) item on the intake form. Because the item did not separately assess suicidal ideation, plans, and attempts, we were not able to differentiate these phenotypes. Nonsuicidal self-injury (NSSI) and unintentional self-harm were not assessed and were not included in the outcome definition. Responses were recorded as binary values (yes/no); No ambiguous responses were recorded.

### Covariates

This study is a secondary analysis of a previously established multicenter outpatient SSD dataset with comprehensive baseline psychometric and laboratory assessments [32]. The available measures were treated as candidate predictors to develop an interpretable machine-learning model for stratifying the likelihood of past-year self-reported suicidality. Covariates were derived from the standardized baseline assessment and included self-report measures, clinician- and self-rated psychological scales, routinely collected laboratory indices, and vital signs. Laboratory variables were included pragmatically because they were already available in this secondary dataset and are readily obtainable in routine outpatient practice. In addition, prior studies have suggested that thyroid dysfunction may be associated with NSSI and suicide attempts among patients with major depressive disorder [33, 34], and lipid abnormalities are closely related to thyroid dysfunction [35]. Therefore, as thyroid function and lipid profiles were collected in the original dataset, we included them as candidate predictors to explore whether they could provide incremental information beyond questionnaire-based measures.

Demographic variables were age (years), sex (female/male), body mass index (BMI) (kg/m²), educational attainment (≥ college vs. < college), and marital status (married vs. other). Clinical characteristics included illness duration (months) and age at onset (years). Psychiatric clinical status comprised depression, anxiety, severe anxiety, and psychotic symptoms (all yes/no). The following scales were entered as continuous raw scores: Hamilton Depression Rating Scale (HAMD) [36], Hamilton Anxiety Rating Scale (HAMA) [37], Patient Health Questionnaire–9 (PHQ-9) [38], Generalized Anxiety Disorder–7 (GAD-7) [39, 40], Insomnia Severity Index (ISI) [41], Rosenberg Self-Esteem Scale (SES) [42], UCLA Loneliness Scale–8 item (ULS-8) [43], Adult Dispositional Hope Scale (ADHS) [44], General Self-Efficacy Scale (GSES) [45], Perceived Stress Scale (PSS) [46], 10-Item Connor–Davidson Resilience Scale (CD-RISC-10) [47], Toronto Alexithymia Scale–20 (TAS-20) [48], Ruminative Responses Scale (RRS) [49], Five Facet Mindfulness Questionnaire (FFMQ) [50], Satisfaction With Life Scale (SWLS) [51], Self-Compassion Scale (SCS) [52], Acceptance and Action Questionnaire–II (AAQ-II) [53], Perceived Social Support Scale (PSSS) [54, 55], Repeatable Battery for the Assessment of Neuropsychological Status (RBANS) [56], Positive and Negative Syndrome Scale (PANSS) [57], and Clinical Global Impression (CGI) [58]. Laboratory covariates included thyroid-stimulating hormone (TSH), free triiodothyronine (FT3), free thyroxine (FT4), anti-thyroglobulin antibody (ATG), anti-thyroid peroxidase antibody (ATPO), fasting plasma glucose (FPG), and lipid profile—total cholesterol (TC), high-density lipoprotein cholesterol (HDL-C), low-density lipoprotein cholesterol (LDL-C), and triglycerides (TG). Vital signs included systolic and diastolic blood pressure (SBP, DBP).

### Statistical analysis and modeling

Data were randomly split into a training set (70%) and a test set (30%). Baseline characteristics were summarized as medians (IQRs) for continuous variables and No. (%) for categorical variables. Distributional assumptions were examined prior to hypothesis testing. Between-group comparisons used the Mann–Whitney U test for continuous variables and the χ² test for categorical variables.

Candidate predictors were screened in the training set using 2 complementary methods—least absolute shrinkage and selection operator (LASSO) regression and the Boruta algorithm. Variables retained for model development were the intersection of features selected by both procedures. Eight algorithms were then trained in the caret framework with 5-fold cross-validation repeated twice: gradient boosting machine (GBM), generalized linear model (GLM), elastic net–regularized logistic regression (GLMNET), random forest (RANGER implementation), support vector machine with radial basis function kernel (SVM), extreme gradient boosting (XGB), naïve Bayes (NB), and a single–hidden-layer neural network (NNET). Model performance was evaluated using the area under the receiver operating characteristic curve (AUC), area under the precision–recall curve (AUPRC), Brier score, accuracy, sensitivity, specificity, positive predictive value (PPV), negative predictive value (NPV), and F1 score. Primary selection metrics were AUC and Brier score. For the top-performing model, we generated calibration plots and conducted decision-curve analysis.

To enhance interpretability, we applied SHAP to the selected model to estimate global feature importance, display summary (beeswarm) plots, produce dependence plots for the 6 highest-contributing features, and create case-level waterfall plots. Finally, we implemented a Shiny-based web calculator to provide individualized risk estimates for past-year self-reported suicidality.

### Interaction assessment

To evaluate whether clinically plausible effect modification materially influenced risk estimation or calibration, we prespecified and tested several interaction terms within a GLM framework. Specifically, we examined the interactions PHQ-9×ISI, PHQ-9×GAD-7, PHQ-9×psychotic symptoms, Sex×PHQ-9, and ULS-8×PSSS. Model fit was compared between the main-effects model and the interaction model using a likelihood ratio test. Discrimination and calibration were evaluated with and without interaction terms by comparing AUROC, Brier score, and calibration parameters (calibration intercept and slope).

### Sensitivity analysis

To assess the robustness of model performance to the choice of data partition, we conducted a sensitivity analysis using 10 repeated random train–test splits with different random seeds. For each split, the full modeling pipeline was repeated and model discrimination was evaluated on the held-out test set by reporting the AUROC and AUPRC for each algorithm. Consistency of AUROC/AUPRC across splits was used to judge the stability of model performance. In addition, to clarify the main drivers of discrimination, we performed ablation analyses using two reduced predictor sets derived from the selected predictors. Specifically, we re-trained and evaluated models using (i) a questionnaire-only set (FFMQ, RBANS, ISI, HAMA, PANSS) and (ii) a laboratory/vital-sign–only set (TSH, ATPO, ATG, BMI, SBP, DBP). These reduced-set analyses were repeated across the same 10 splits, and test-set AUROC and AUPRC were reported to compare performance stability and to assess the relative contribution of psychometric versus laboratory/vital-sign information.

## Results

### Characteristics of the study population

As shown in Table 1, among 899 participants (median age, 33 years [IQR, 24–45]; 64.4% female), median illness duration was 11 months (IQR, 9–14) and age at onset was 32 years (IQR, 23–45). Educational attainment was ≥college in 33.6%, and 67.6% were married. Suicidal attempt was present in 19.9%. Depressive and anxiety symptoms were common (47.2% and 77.5%, respectively); severe anxiety occurred in 12.4% and psychotic symptoms in 10.2%.

**Table 1:** Baseline characteristics of the train and test sets

	All *n* = 899	Test *n* = 269	Train *n* = 630	*P*
Age (year)	33.00 (24.00,45.00)	34.00 (24.00,47.00)	32.00 (24.00,44.00)	0.161
Duration (month)	11.00 (9.00,14.00)	11.00 (9.00,14.00)	11.00 (9.00,14.00)	0.351
Onset age (year)	32.00 (23.00,45.00)	34.00 (24.00,47.00)	32.00 (23.00,44.00)	0.146
Female	579 (64.40%)	178 (66.17%)	401 (63.65%)	0.518
BMI (kg/m^2^)	24.21 (23.15,25.60)	24.18 (23.21,25.60)	24.22 (23.15,25.60)	0.934
Education				0.659
≥college	302 (33.59%)	87 (32.34%)	215 (34.13%)	
< college	597 (66.41%)	182 (67.66%)	415 (65.87%)	
Marital				0.578
married	608 (67.63%)	186 (69.14%)	422 (66.98%)	
other	291 (32.37%)	83 (30.86%)	208 (33.02%)	
Suicidal behavior				0.991
no	720 (80.09%)	216 (80.30%)	504 (80.00%)	
yes	179 (19.91%)	53 (19.70%)	126 (20.00%)	
Severe anxiety				0.776
no	788 (87.65%)	234 (86.99%)	554 (87.94%)	
yes	111 (12.35%)	35 (13.01%)	76 (12.06%)	
Psychotic symptoms				0.475
no	807 (89.77%)	238 (88.48%)	569 (90.32%)	
yes	92 (10.23%)	31 (11.52%)	61 (9.68%)	
Depression				0.957
no	475 (52.84%)	143 (53.16%)	332 (52.70%)	
yes	424 (47.16%)	126 (46.84%)	298 (47.30%)	
Anxiety				0.291
no	202 (22.47%)	67 (24.91%)	135 (21.43%)	
yes	697 (77.53%)	202 (75.09%)	495 (78.57%)	
HAMD	23.00 (21.00,25.00)	23.00 (21.00,25.00)	23.00 (21.00,25.00)	0.842
HAMA	21.00 (18.00,23.00)	20.00 (17.00,23.00)	21.00 (18.00,23.00)	0.511
PHQ-9	11.00 (5.00,18.00)	11.00 (5.00,18.00)	11.00 (5.00,18.00)	0.538
GAD-7	10.00 (10.00,15.00)	10.00 (8.00,17.00)	10.00 (10.00,15.00)	0.951
ISI	14.00 (8.00,21.00)	14.00 (8.00,21.00)	14.00 (8.00,21.00)	0.716
Sleep duration (hour)	5.50 (4.50,6.50)	5.50 (4.50,6.50)	5.50 (4.50,6.50)	0.576
SES	20.00 (10.00,30.00)	20.00 (10.00,30.00)	20.00 (10.00,30.00)	0.845
ULS-8	24.00 (17.00,29.00)	24.00 (17.00,29.00)	24.00 (17.00,29.00)	0.960
ADHS	19.00 (17.00,24.00)	19.00 (17.00,25.00)	19.00 (17.00,24.00)	0.929
GSES	13.00 (11.00,22.00)	13.00 (11.00,24.00)	13.00 (11.00,21.00)	0.562
PSS	30.00 (22.00,34.00)	30.00 (20.00,34.00)	30.00 (22.00,34.00)	0.922
CD-RISC-10	13.00 (7.00,20.00)	13.00 (7.00,20.00)	13.00 (7.00,20.00)	0.769
TAS-20	79.00 (66.00,83.00)	79.00 (66.00,83.00)	79.00 (66.00,83.00)	0.853
RRS	69.00 (48.00,88.00)	69.00 (42.00,88.00)	69.00 (48.00,88.00)	0.862
FFMQ	133.00 (127.00,134.00)	133.00 (127.00,134.00)	133.00 (127.00,134.00)	0.452
SWLS	12.00 (7.00,15.00)	12.00 (7.00,17.00)	12.00 (7.00,15.00)	0.635
SCS	27.00 (19.00,36.00)	27.00 (19.00,39.00)	25.00 (19.00,36.00)	0.766
AAQ-II	37.00 (31.00,39.00)	37.00 (26.00,40.00)	37.00 (31.00,39.00)	0.779
PSSS	36.00 (20.00,58.00)	36.00 (20.00,58.00)	36.00 (20.00,58.00)	0.886
RBANS	68.00 (62.00,83.00)	69.00 (64.00,83.00)	68.00 (62.00,83.00)	0.224
PANSS	7.00 (7.00,7.00)	7.00 (7.00,8.00)	7.00 (7.00,7.00)	0.581
CGI	6.00 (5.00,7.00)	6.00 (5.00,7.00)	6.00 (5.00,7.00)	0.503
TSH (mIU/L)	4.88 (3.03,6.62)	4.81 (2.89,6.59)	4.91 (3.12,6.68)	0.743
ATG (IU/mL)	21.27 (14.42,42.64)	20.86 (13.85,29.39)	21.30 (14.58,52.45)	0.091
ATPO (IU/mL)	17.19 (12.25,33.56)	16.97 (12.23,29.21)	17.37 (12.27,35.20)	0.392
FT3 (pmol/L)	4.90 (4.38,5.40)	4.86 (4.41,5.32)	4.94 (4.36,5.41)	0.502
FT4 (pmol/L)	16.58 (14.38,18.80)	16.75 (14.76,18.87)	16.41 (14.30,18.70)	0.103
FPG (mmol/L)	5.31 (4.90,5.77)	5.31 (4.92,5.81)	5.31 (4.90,5.76)	0.346
TC (mmol/L)	5.14 (4.42,5.92)	5.12 (4.40,5.87)	5.18 (4.43,5.96)	0.700
HDLC (mmol/L)	1.23 (1.01,1.42)	1.25 (1.01,1.46)	1.22 (1.01,1.41)	0.307
TG (mmol/L)	1.96 (1.37,2.78)	2.03 (1.41,2.80)	1.90 (1.36,2.76)	0.330
LDLC (mmol/L)	2.90 (2.30,3.50)	2.85 (2.30,3.50)	2.90 (2.31,3.42)	0.950
SBP (mmHg)	120.00 (112.00,127.00)	120.00 (112.00,128.00)	120.00 (112.00,126.00)	0.390
DBP (mmHg)	76.00 (70.50,80.00)	76.00 (70.00,80.00)	76.00 (71.00,80.00)	0.841

Data are presented as median (Q1, Q3) or n (%). Q1, 1st Quartile; Q3, 3rd Quartile; BMI, body mass index; HAMD, Hamilton Depression Scale; HAMA, Hamilton Anxiety Scale; PHQ-9, Patient Health Questionnaire-9 items; GAD-7, Generalized Anxiety Disorder-7; ISI, Insomnia Severity Index; SES, Rosenberg Self-Esteem Scale; ULS-8, UCLA Loneliness Scale; ADHS, Adult Dispositional Hope Scale; GSES, General Self-Efficacy Scale; PSS, Perceived Stress Scales; CD-RISC-10, 10-Item Connor-Davidson Resilience Scale; TAS-20, Toronto Alexithymia Scale-20; RRS, Ruminative Responses Scale; FFMQ, The Five Facet Mindfulness Questionnaire; SWLS, Satisfaction with Life Scale; SCS, Self-Compassion Scale; AAQ-II, Acceptance and Action Questionnaire-II; PSSS, Perceived Social Support Scale; RBANS, The repeatable battery for the assessment of neuropsychological status; PANSS, Positive and Negative Syndrome; CGI, Clinical Global Impression; TSH, Thyroid-Stimulating Hormone; ATG, Anti-Thyroglobulin Antibody; ATPO, Anti-Thyroid Peroxidase Antibody; FT3, Free Triiodothyronine; FT4, Free Thyroxine; FPG, Fasting Plasma Glucose; TC, Total Cholesterol; HDLC, High-Density Lipoprotein Cholesterol; TG, Triglycerides; LDLC, Low-Density Lipoprotein Cholesterol; SBP, Systolic Blood Pressure; DBP, Diastolic Blood Pressure

Participants were randomly allocated to the training (*n* = 630) and test (*n* = 269) sets. Baseline demographic, clinical, psychometric, laboratory, and vital-sign characteristics were well balanced between the two sets, and no statistically significant differences were observed across variables (all *P* > 0.05) (Table 1).

### Variable selection

**Fig. 1:**
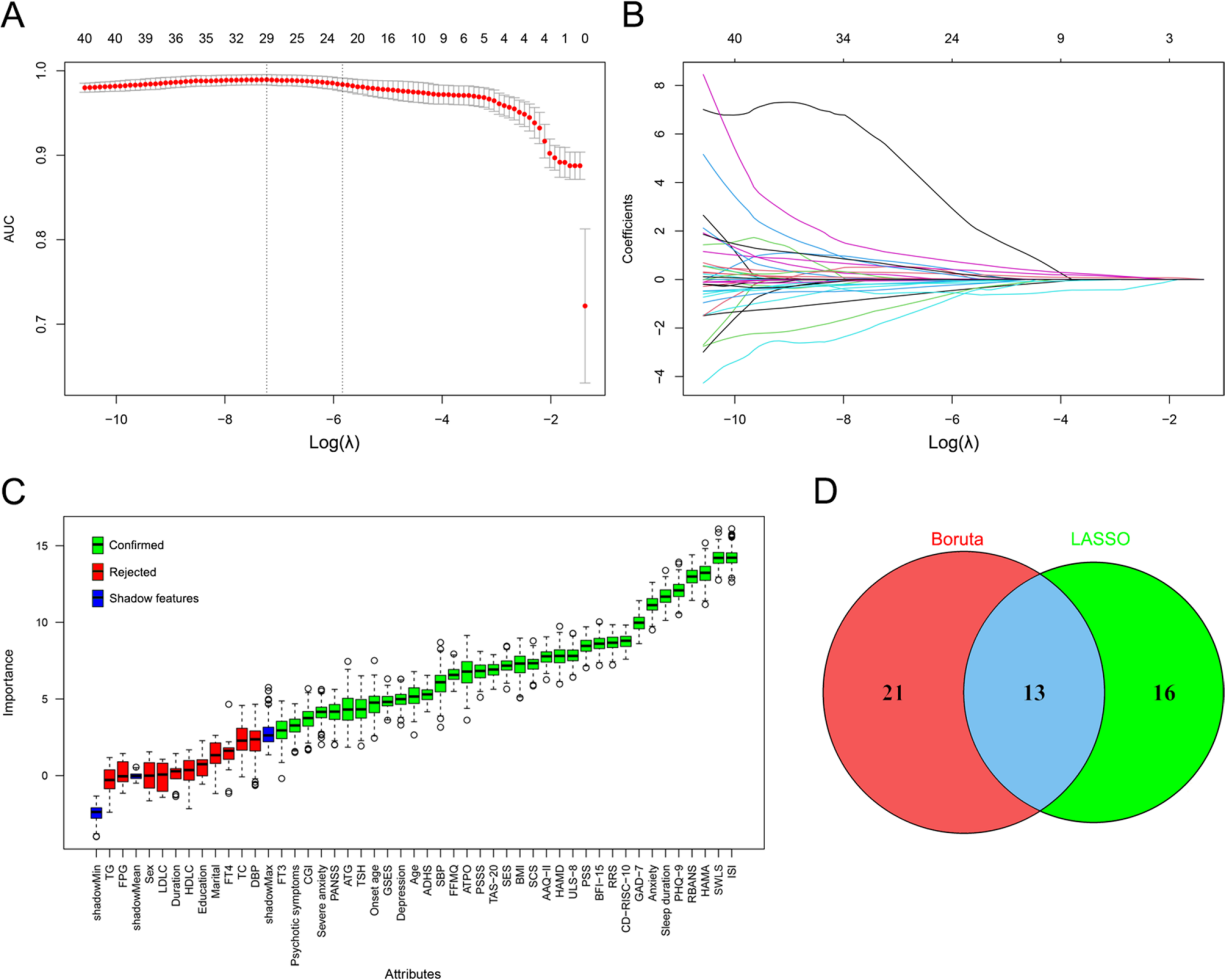
Variable selection using LASSO and Boruta. **A**, Cross-validated performance of LASSO. **B**, LASSO coefficient paths across the penalty grid. **C**, Boruta feature importance. **D**, Overlap of predictors retained by Boruta and LASSO. Abbreviations: AUC, area under the curve; LASSO, least absolute shrinkage and selection operator; λ, lambda; other abbreviations are defined in methods

**Fig. 2:**
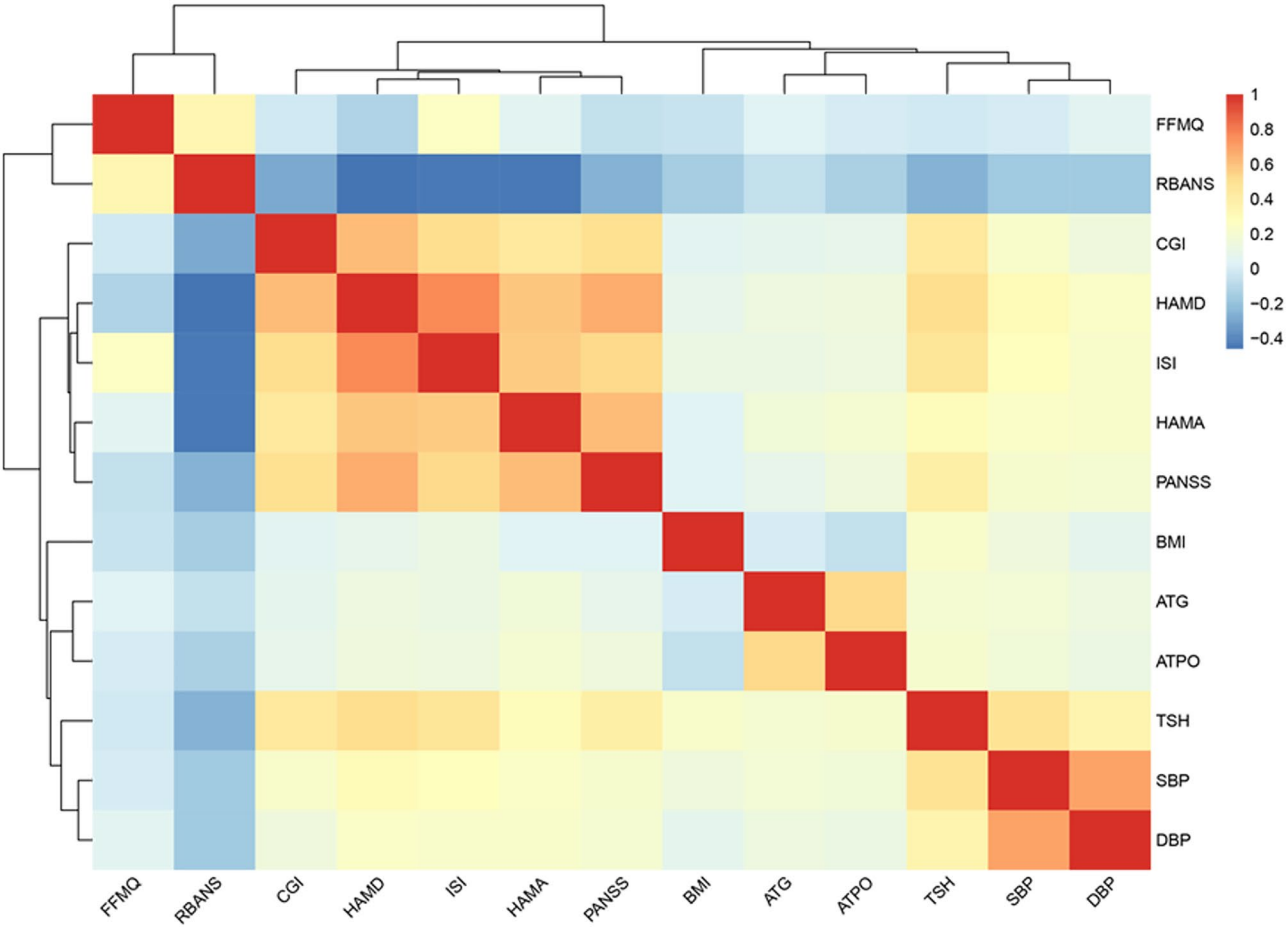
Correlation heatmap of candidate predictors. Heatmap shows pairwise Spearman correlation coefficients among candidate predictors; colors indicate direction and magnitude (blue, negative; red, positive). Dendrograms display hierarchical clustering of variables. Abbreviations are defined in Methods

Using LASSO and Boruta, we identified 29 and 34 candidate variables associated with past-year self-reported suicidality, respectively; their intersection yielded 13 variables (Fig. 1A–D). ISI and HAMD were highly correlated (*r* > 0.70), indicating collinearity (Fig. 2). Because HAMD contains suicide-related content that may overlap with the outcome, we excluded HAMD to reduce potential circularity. In addition, CGI represents clinician-rated global illness severity and may partially reflect clinicians’ awareness of suicidality; therefore, CGI was removed to mitigate potential information leakage. Accordingly, 11 variables were retained for model development: FFMQ, RBANS, ISI, HAMA, PANSS, BMI, ATG, ATPO, TSH, SBP, and DBP.

### Model development and performance

**Fig. 3:**
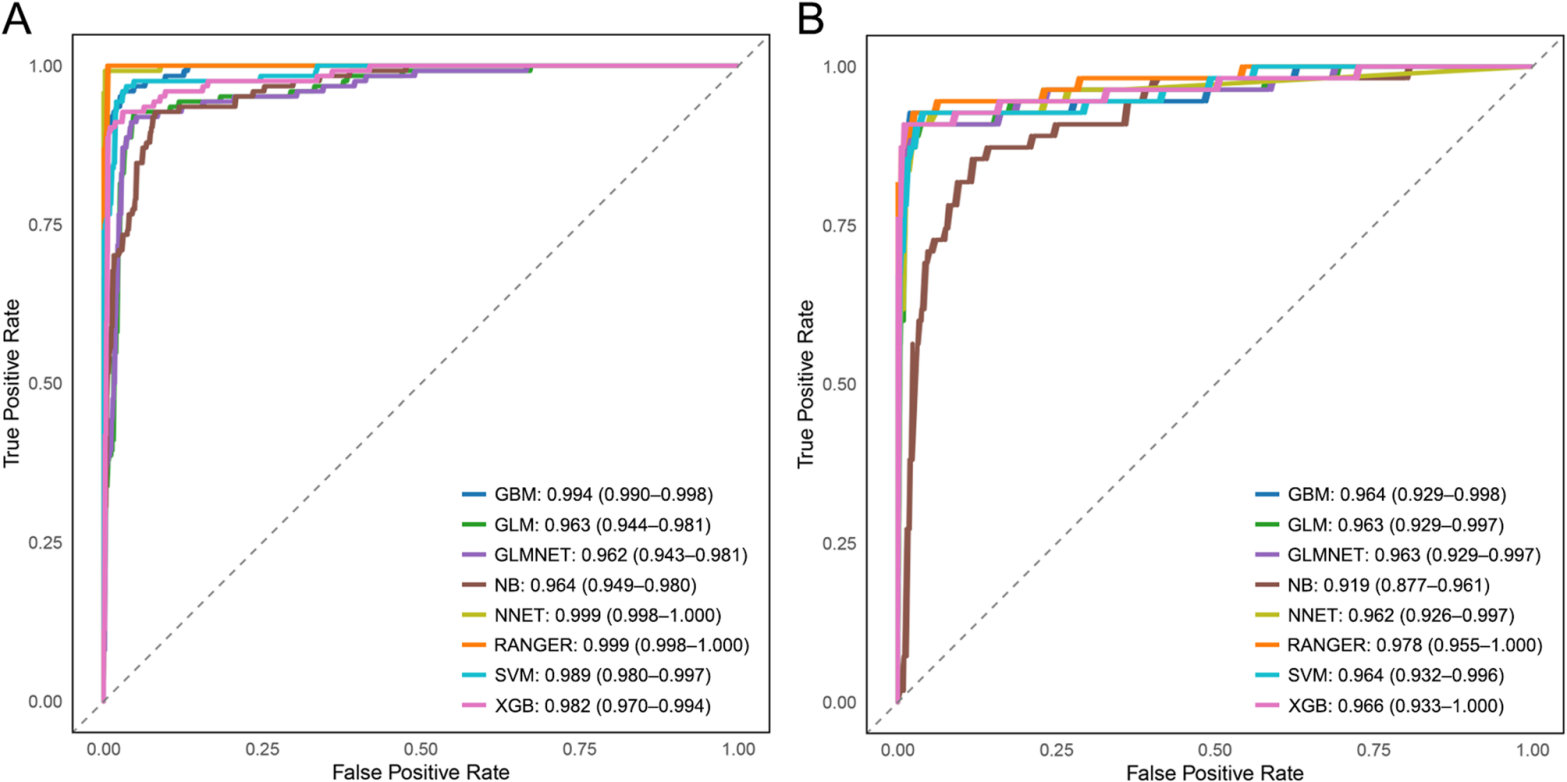
Receiver operating characteristic (ROC) curves for model discrimination in Train and Test sets. **A**, Train set. **B**, Test set. AUC (95% CI) for each algorithm is shown in the in-figure legend. Abbreviations: AUC, area under the ROC curve; GBM, gradient boosting machine; GLM, generalized linear model; GLMNET, elastic-net regularized logistic regression; NB, naïve Bayes; NNET, single–hidden-layer neural network; RANGER, random forest; SVM, support vector machine (radial basis); XGB, extreme gradient boosting

**Fig. 4:**
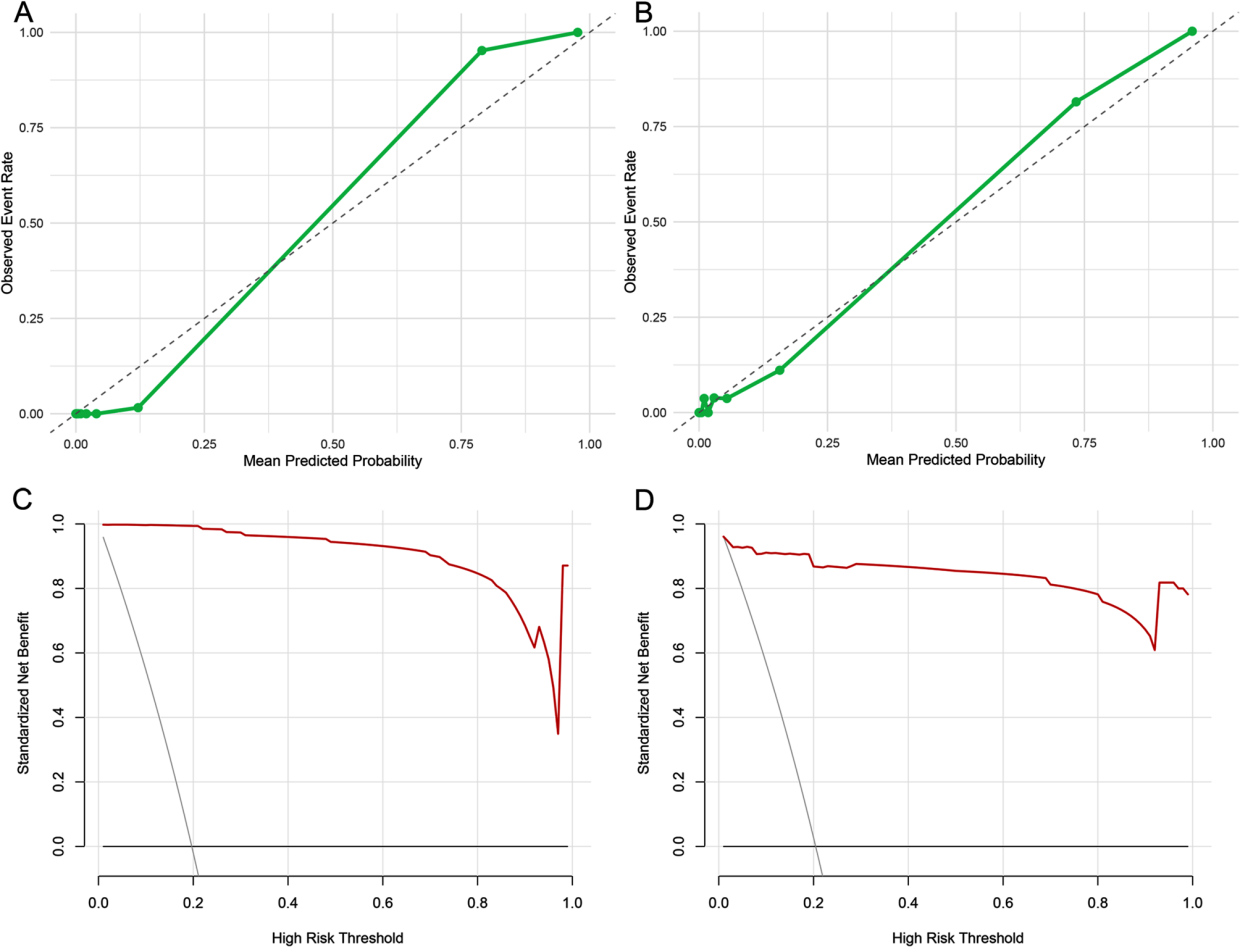
Calibration and Decision-curve analysis for the RANGER model in Train and Test sets. **A**–**B**, Calibration. Observed event rates vs. mean predicted probabilities for RANGER; dashed line indicates perfect calibration. **A**, Training set. **B**, Test set. **C**–**D**, Decision-curve analysis. Standardized net benefit across threshold probabilities; red curve, RANGER; dark gray, treat-all; black, treat-none. **C**, Training set. **D**, Test set. RANGER, random forest

ROC curves showed high discrimination across models (Fig. 3A–B). In the test set, RANGER achieved the highest AUROC (0.978; 95% CI, 0.955–1.000), followed by XGB (0.966; 95% CI, 0.933–1.000), GBM (0.964; 95% CI, 0.929–0.998) and SVM (0.964; 95% CI, 0.932–0.996), with GLM/GLMNET both showing AUROC of 0.963 (95% CI, 0.929–0.997), and NNET AUROC of 0.962 (95% CI, 0.926–0.997); NB showed comparatively lower AUROC (0.919; 95% CI, 0.877–0.961). Training-set AUROCs were generally higher, particularly for RANGER and NNET (Fig. 3A). Consistent with AUROC results, the performance metrics in the test set indicated that RANGER had the highest AUPRC (0.960) and the lowest Brier score (0.028), with high overall accuracy (0.967) and balanced sensitivity (0.927) and specificity (0.977) (Table 2). Calibration plots indicated good agreement between predicted and observed risk, and decision-curve analysis suggested net benefit across a broad range of clinically relevant threshold probabilities compared with treat-all and treat-none strategies (Fig. 4A–D). Two prespecified interactions (PHQ-9×ISI and PHQ-9×psychotic symptoms) were statistically significant, whereas including interaction terms resulted in only minor improvement in discrimination and no meaningful change in calibration (Table S1, S2). Sensitivity analyses using 10 different random seeds for repeated train–test splits showed stable model performance: test-set AUROC (and AUPRC) estimates were highly consistent across splits, indicating that the results were robust to the choice of random partition (Table S3). In additional ablation analyses, models restricted to the questionnaire-only predictor set retained strong discrimination across the 10 repeated splits (test-set AUROC consistently > 0.90 with generally high AUPRC) (Table S4). In contrast, models using laboratory and vital-sign variables only showed weaker but still moderate discrimination (test-set AUROC ~ 0.66–0.80 with modest AUPRC) (Table S5).

**Table 2:** Performance metrics of machine learning models in the train and test sets

Model	Split	AUPRC	Brier	Threshold	Accuracy	Sensitivity	Specificity	PPV	NPV	F1
GBM	Train	0.978	0.023	0.233	0.970	0.952	0.974	0.901	0.988	0.925
	Test	0.945	0.034	0.233	0.970	0.927	0.981	0.927	0.981	0.927
GLM	Train	0.881	0.049	0.367	0.946	0.903	0.957	0.836	0.976	0.868
	Test	0.932	0.039	0.367	0.955	0.909	0.967	0.877	0.976	0.893
GLMNET	Train	0.880	0.050	0.412	0.946	0.895	0.958	0.841	0.974	0.867
	Test	0.936	0.040	0.412	0.963	0.909	0.977	0.909	0.977	0.909
RANGER	Train	0.997	0.011	0.269	0.994	1.000	0.992	0.969	1.000	0.984
	Test	0.960	0.028	0.269	0.967	0.927	0.977	0.911	0.981	0.919
SVM	Train	0.970	0.025	0.317	0.967	0.952	0.970	0.887	0.988	0.918
	Test	0.937	0.037	0.317	0.955	0.927	0.963	0.864	0.981	0.895
XGB	Train	0.942	0.030	0.344	0.967	0.911	0.980	0.919	0.978	0.915
	Test	0.948	0.033	0.344	0.974	0.909	0.991	0.962	0.977	0.935
NB	Train	0.890	0.059	0.045	0.894	0.927	0.885	0.665	0.980	0.774
	Test	0.709	0.088	0.045	0.877	0.855	0.883	0.653	0.959	0.740
NNET	Train	0.998	0.003	0.182	0.997	0.992	0.998	0.992	0.998	0.992
	Test	0.934	0.040	0.182	0.959	0.909	0.972	0.893	0.977	0.901

AUPRC, area under the precision–recall curve; PPV, positive predictive value; NPV, negative predictive value; GBM, gradient boosting machine; GLM, generalized linear model (logistic regression); GLMNET, generalized linear model with elastic-net regularization; RANGER, random forest (ranger implementation); SVM, support vector machine; XGB, extreme gradient boosting; NB, naive Bayes; NNET, feed-forward neural network

### Model explanation

**Fig. 5:**
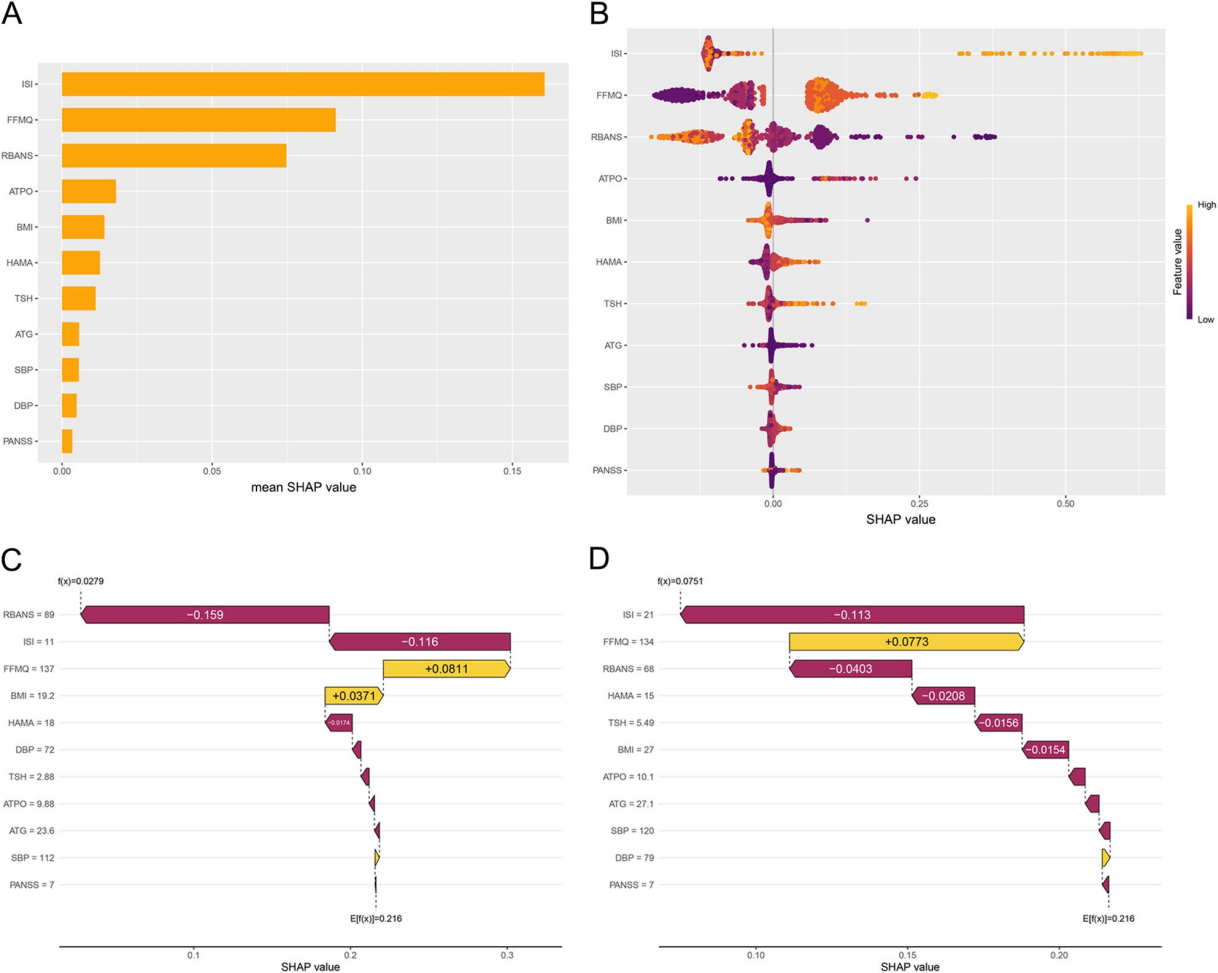
SHAP Explanations for the RANGER Model. **A**, Global feature importance ranked by mean absolute SHAP value. **B**, Beeswarm plot showing feature-wise SHAP values and directionality. **C**, Case-level waterfall plot for a high-risk prediction. **D**, Case-level waterfall plot for a low-risk prediction. Abbreviations: SHAP, Shapley additive explanations; RANGER, random forest. Other variable abbreviations are defined in Methods

**Fig. 6:**
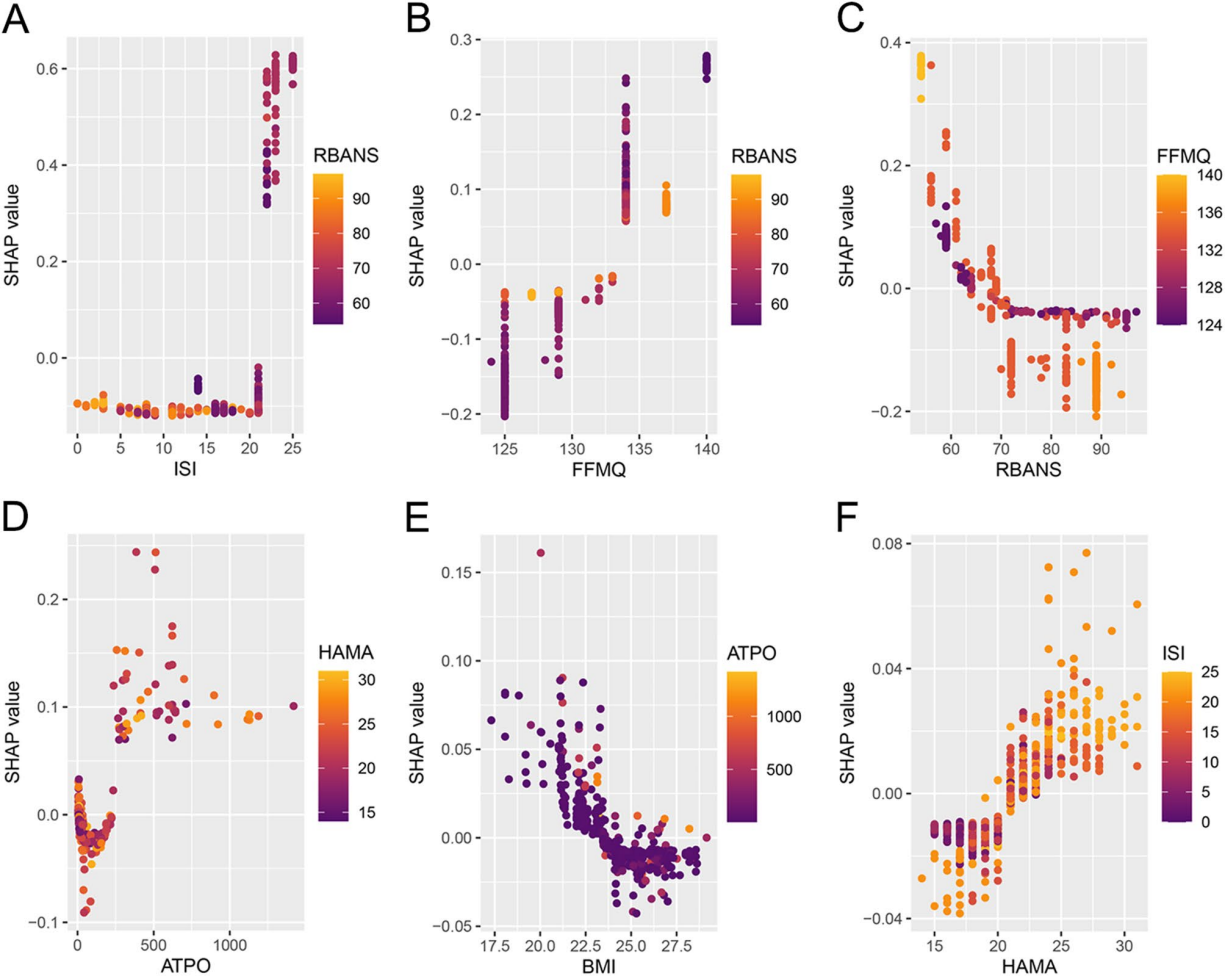
SHAP Dependence Plots for the RANGER Model. Panels show feature-wise SHAP dependence with color indicating interacting feature values. **A**, ISI (color = RBANS). **B**, FFMQ (color = RBANS). **C**, RBANS (color = FFMQ). **D**, ATPO (color = HAMA). **E**, BMI (color = ATPO). **F**, HAMA (color = ISI). Abbreviations: SHAP, Shapley additive explanations; RANGER, random forest. Other variable abbreviations are defined in Methods

In the RANGER model, SHAP feature-importance rankings identified ISI as the most influential predictor, followed by FFMQ and RBANS, with smaller contributions from ATPO, BMI, and HAMA (Fig. 5A). The SHAP beeswarm plot suggested clear directionality: higher ISI and HAMA were associated with increased predicted risk of past-year self-reported suicidality, whereas higher RBANS was associated with decreased risk; higher FFMQ values generally corresponded to higher predicted risk (Fig. 5B). Individual-level waterfall plots illustrated how combinations of these features shifted predictions from the baseline risk (Fig. 5C–D). Dependence plots further indicated a threshold-like increase in SHAP values at higher ISI scores (approximately in the low-to-mid 20s) and a similar threshold pattern for FFMQ, an approximately monotonic inverse association for RBANS, and a positive association for HAMA (Fig. 6A–C and F). ATPO and BMI exhibited nonlinear relationships with predicted risk (Fig. 6D–E). Color gradients suggested modest interactions, for example, the contribution of ISI/FFMQ appeared more pronounced when RBANS was lower, and the contribution of HAMA tended to be larger when ISI was higher (Fig. 6A–B and F).

### Web-based risk calculator

**Fig. 7:**
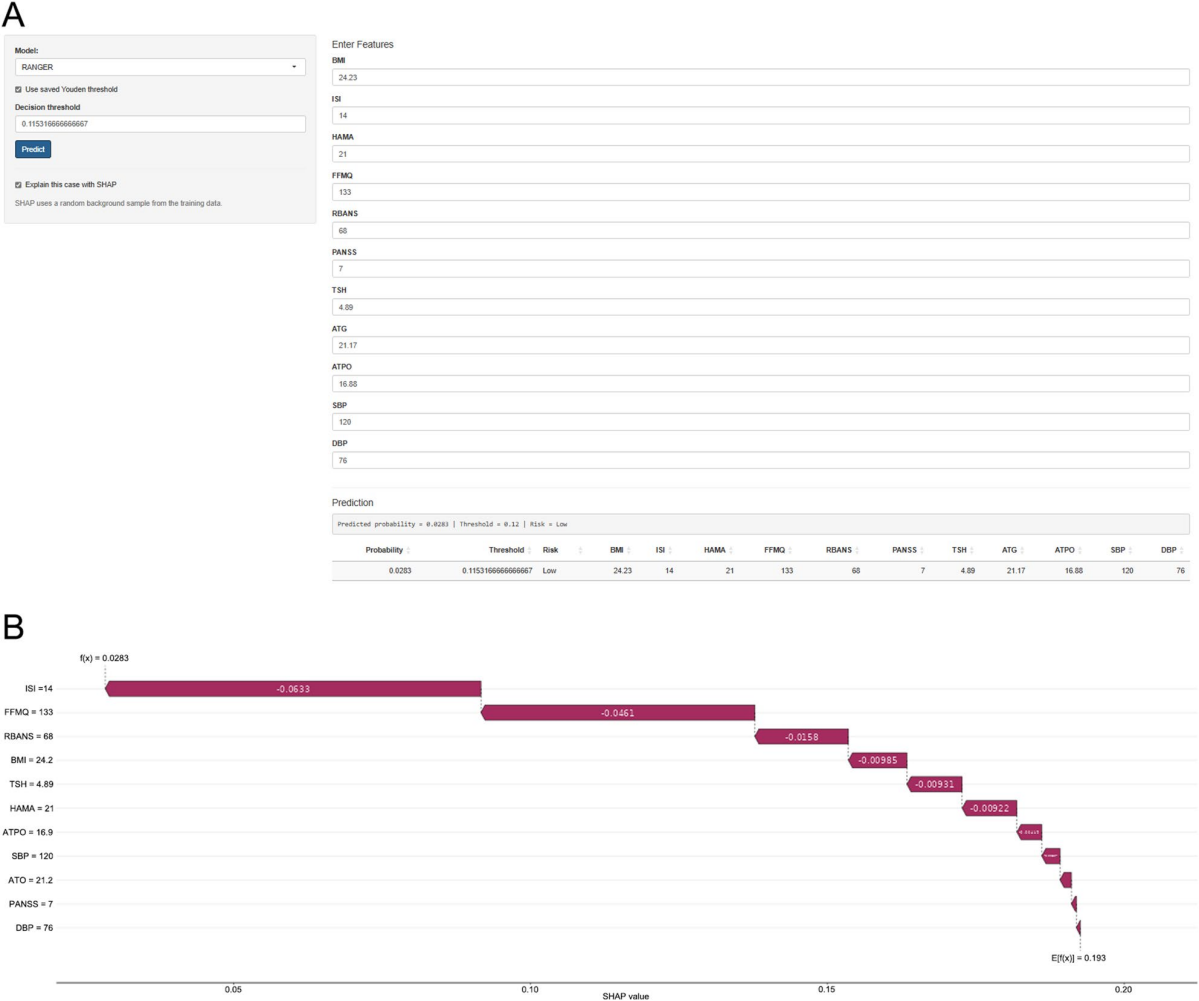
Web-Based Risk Calculator for Outpatients With Somatic Symptom Disorder. Screenshots of the Shiny-deployed calculator. A, Interface without SHAP enabled: users select the model (e.g., RANGER), enter values for the 11 predictors, and obtain the predicted probability and risk category. B, Interface with SHAP enabled: an individualized waterfall plot displays the direction and magnitude of each predictor’s contribution to the prediction. Abbreviations: SHAP, Shapley additive explanations; RANGER, random forest. Other variable abbreviations are defined in Methods

We deployed all eight algorithms as a Shiny-based web calculator (available at https://zts1994neurology.shinyapps.io/SSD-suicide-risk1/). Users can select any of the eight models, enter values for the 11 predictors used in model development, and obtain the predicted probability of past-year self-reported suicidality (Fig. 7A). When the SHAP explanation option is enabled, the tool also returns an individualized waterfall plot showing the direction and magnitude of each predictor’s contribution (Fig. 7B). The calculator is provided for research/educational use pending external validation.

## Discussion

In this multicenter registry of adults with SSD, we developed interpretable machine-learning models to classify participants who reported a past-year self-reported suicidality. The RANGER model showed the best overall internal performance with good calibration. SHAP analyses indicated that ISI was the most influential contributor, followed by FFMQ and RBANS. These findings extend prior work by showing that a multidomain, interpretable approach can support screening and stratification for past-year self-reported suicidality within SSD care pathways, a context in which suicide-risk assessment may be overlooked because patients present with prominent somatic complaints. The accompanying web-based calculator illustrates potential clinical utility, although external validation and prospective evaluation are needed before routine implementation.

Prior predictive models of suicidal behavior have rarely targeted outpatients with SSD, although numerous studies have examined other psychiatric populations. Deep-learning analyses of structural magnetic resonance imaging in major depressive disorder (MDD) have been used to identify suicide risk, and multimodal studies have related brain functional and structural metrics to nonsuicidal self-injury in MDD [59, 60]. Other work has leveraged blood methylome and transcriptome data to predict risks of MDD and suicidal behavior [61]. These approaches advance understanding but are difficult to operationalize in routine care because such data are not typically available in outpatient settings. In contrast, Saeed et al. used readily obtainable information—standardized symptom scales and thyroid function tests—to build a suicide-risk model for bipolar disorder, highlighting depressive symptoms and thyroid function as key correlates; however, usability remained limited [62]. In the present study, we implemented a Shiny-based web calculator that can be accessed on a mobile device and relies on inputs commonly available during outpatient visits (questionnaire scores, basic laboratory tests, and vital signs). The models demonstrated good performance, with test-set AUCs exceeding 0.95 across algorithms, alongside favorable results on secondary metrics.

Consistent with prior work, ISI and HAMA showed positive contributions to suicide risk, reinforcing sleep disturbance and anxiety as clinically actionable correlates in outpatient settings [63–65]. Higher cognitive performance (RBANS) was inversely associated with risk, aligning with reports that cognitive dysfunction is linked to suicidal behaviors in mood and psychotic disorders [66, 67]. The positive contribution of FFMQ was unexpected; possible explanations include construct overlap with ruminative attention in highly somatically focused patients, differential functioning of specific FFMQ subscales, or residual confounding. ATPO exhibited a small, nonlinear association, compatible with mixed evidence that subclinical thyroid dysfunction may relate to mood dysregulation and suicidality [68, 69]. BMI showed a modest, nonlinear pattern, echoing literature that extremes of weight can co-occur with sleep disturbance, anxiety, and somatic symptom burden [70]. Overall, these patterns corroborate established risk domains (sleep, anxiety, cognition) while highlighting potentially context-specific signals (mindfulness facets, thyroid autoantibodies) in SSD. Taken together, the feature profile suggests readily assessable targets for risk triage in SSD pathways where suicidality may be overlooked. Sleep and anxiety metrics (ISI, HAMA) can be integrated into routine screening, whereas lower cognitive scores (RBANS) may flag individuals who need closer monitoring and safety planning. Signals in FFMQ and ATPO warrant cautious interpretation and replication; they may capture somatic hypervigilance or immune-endocrine vulnerability in a subset of patients. Because SHAP quantifies associations within a predictive framework rather than causal effects, these findings should guide hypothesis generation and pragmatic care (e.g., addressing insomnia and anxiety), while prospective studies evaluate whether modifying these domains reduces risk.

To better understand the unusually high discrimination and to clarify which data domains mainly drive performance, we conducted prespecified ablation analyses across the same 10 repeated train–test splits. Models restricted to a questionnaire-only subset (FFMQ, RBANS, ISI, HAMA, PANSS) consistently retained strong test-set discrimination (AUROC generally > 0.90 with high AUPRC), indicating that proximal symptom burden and cognitive/affective profiles captured most of the predictive signal in this cross-sectional setting. In contrast, models using laboratory and vital-sign variables only (TSH, ATPO, ATG, BMI, SBP, DBP) yielded weaker but still moderate discrimination (test-set AUROC approximately 0.66–0.80 with modest AUPRC), suggesting that routinely collected physiological measures carry incremental—albeit limited—information beyond questionnaires. These findings provide a plausible explanation for the high overall AUROC: the strongest signals likely reflect contemporaneous clinical severity (e.g., insomnia and anxiety) that may be proximal to, or partly shaped by, past-year suicidality rather than representing antecedent risk. Accordingly, our tool is best interpreted as a pragmatic outpatient triage aid to flag patients who warrant prioritized suicide-risk assessment, rather than as a model for forecasting future suicidal behavior. Among non-questionnaire features, thyroid autoantibodies (ATPO) and blood pressure were retained after feature selection and showed modest contributions, supporting further investigation of immune–endocrine and autonomic/cardiometabolic correlates of past-year suicidality in SSD, ideally in prospective and externally validated cohorts.

This study has several strengths: (1) a multicenter outpatient cohort of individuals with SSD, addressing a population in which suicide risk may be underrecognized; (2) rigorous variable selection using complementary methods (LASSO and Boruta) with prespecified criteria for model choice; (3) comprehensive performance assessment including discrimination, calibration, and decision-analytic net benefit rather than AUC alone; (4) transparent model explainability with SHAP at both global and individual levels; and (5) a pragmatic, Shiny-based web calculator that enables bedside use with routinely obtainable inputs.

This work also had limitations. First, the cross-sectional design precluded causal inference and did not capture the timing, recency, or recurrence of suicidality episodes; reverse causality cannot be excluded, as current symptom severity may partly reflect prior attempts rather than antecedent risk. Second, the primary outcome was derived from a single questionnaire item, introducing potential measurement error and misclassification; the assessment did not capture severity, frequency, recency, or other suicidal phenotypes. Third, although internal validation was strong, the cohort included adults aged 18–60 years from 3 hospitals in one region and one ethnic group, which may limit generalizability. In addition, source hospital identifiers were not retained in the analytic dataset and access to full outpatient records was restricted to protect privacy; therefore, site-specific external validation could not be performed. Fourth, residual confounding is possible (e.g., unmeasured psychosocial or treatment variables). Fifth, although we excluded outcome-adjacent measures (e.g., CGI and HAMD) to mitigate potential information leakage, some predictors may still represent proximal correlates of prior attempts, which could inflate internal performance. Sixth, restricting feature selection to the intersection of LASSO and Boruta may have excluded weak but stable predictors.

## Conclusion

In a multicenter cross-sectional registry of individuals with SSD, a RANGER model stratified the likelihood of past-year self-reported suicidality with good discrimination, favorable calibration, and net clinical benefit. Model explainability highlighted insomnia, anxiety, and cognition as prominent contributors to model-based risk stratification. Although the web-based calculator illustrates potential utility, external validation and prospective implementation studies are warranted before routine adoption.

## Supplementary Information

Below is the link to the electronic supplementary material.

Supplementary Material 1

Supplementary Material 2

Supplementary Material 3

Supplementary Material 4

Supplementary Material 5

## Data Availability

The data that support the finding of this study are not openly available due to reasons of sensitivity and are available from the corresponding author upon reasonable request (Zhuts1994@163.com). The analysis code is available in the Supplementary Materials.

## References

[1] Links PS, Aslam H, O’Donnell M. Personality disorders and clinical disorders: the challenge of comorbid autism spectrum disorder (ASD), eating disorders (EDs), posttraumatic stress disorder (PTSD), or somatic symptom disorder (SSD). Curr Psychiat Rep. 2025;27(1):1–9. 10.1007/s11920-024-01571-8 39607574

[2] Wu H, Manglike A, Chen Y, Liu Z, Fritzsche K, Lu Z. Scoping review update on somatic symptom disorder that includes additional Chinese data. Gen Psychiat. 2023;36(3):e100942. 10.1136/gpsych-2022-100942 PMC10277133 37337547

[3] Lowe B, Levenson J, Depping M, Husing P, Kohlmann S, Lehmann M, Shedden-Mora M, Toussaint A, Uhlenbusch N, Weigel A. Somatic symptom disorder: a scoping review on the empirical evidence of a new diagnosis. Psychol Med. 2022;52(4):632–48. 34776017 10.1017/S0033291721004177 PMC8961337

[4] Barsky AJ, Orav EJ, Bates DW. Somatization increases medical utilization and costs independent of psychiatric and medical comorbidity. Arch Gen Psychiatry. 2005;62(8):903–10. 16061768 10.1001/archpsyc.62.8.903

[5] Seo JH, Han M, Kang S, Kim SJ, Jung I, Kang JI. Healthcare utilization and costs in patients with somatic symptom and related disorders compared with those with depression and healthy controls: a nationwide cohort study. Depress Anxiety. 2024;2024:8352965. 10.1155/da/8352965 PMC11918987 40226752

[6] Nazzal Z, Maraqa B, Abu Zant M, Qaddoumi L, Abdallah R. Somatic symptom disorders and utilization of health services among Palestinian primary health care attendees: a cross-sectional study. BMC Health Serv Res. 2021;21(1):615. 34182995 10.1186/s12913-021-06671-2 PMC8240383

[7] Torres ME, Lowe B, Schmitz S, Pienta JN, Van Der Feltz-Cornelis C, Fiedorowicz JG. Suicide and suicidality in somatic symptom and related disorders: A systematic review. J Psychosom Res. 2021;140:110290. 33227556 10.1016/j.jpsychores.2020.110290 PMC7945369

[8] Vassilopoulos A, Poulopoulos L, Ibeziako N. School absenteeism as a potential proxy of functionality in pediatric patients with somatic symptom and related disorders. Clin Child Psychol P. 2021;26(2):342–54. 10.1177/1359104520978462 33287565

[9] Lim K, Wong CH, McIntyre RS, Wang J, Zhang Z, Tran BX, Tan W, Ho CS, Ho RC. Global lifetime and 12-Month prevalence of suicidal Behavior, deliberate Self-Harm and Non-Suicidal Self-Injury in children and adolescents between 1989 and 2018: A Meta-Analysis. Int J Environ Res Public Health. 2019;16(22):4581. 31752375 10.3390/ijerph16224581 PMC6888476

[10] Morales-Arjona I, Benítez-Hidalgo V, Ruiz-Pérez I, Higueras-Callejón C, Pastor-Moreno G. Cyberbullying and suicidal Behavior, Self-Harm, and nonsuicidal Self-Injury: A systematic review of longitudinal studies. Cyberpsychology Behav Social Netw. 2024;27(10):683–91. 10.1089/cyber.2024.0097 39207253

[11] Rosendal M, Olde Hartman TC, Aamland A, van der Horst H, Lucassen P, Budtz-Lilly A, Burton C. Medically unexplained symptoms and symptom disorders in primary care: prognosis-based recognition and classification. BMC Fam Pract. 2017;18(1):18. 28173764 10.1186/s12875-017-0592-6 PMC5297117

[12] Löwe B, Toussaint A, Rosmalen JGM, Huang W, Burton C, Weigel A, Levenson JL, Henningsen P. Persistent physical symptoms: definition, genesis, and management. Lancet (London England). 2024;403(10444):2649–62. 38879263 10.1016/S0140-6736(24)00623-8

[13] Wiborg JF, Gieseler D, Löwe B. Suicidal ideation in German primary care. Gen Hosp Psychiat. 2013;35(4):366–9. 10.1016/j.genhosppsych.2013.02.001 23473475

[14] Torres ME, Löwe B, Schmitz S, Pienta JN, Van Der Feltz-Cornelis C, Fiedorowicz JG. Suicide and suicidality in somatic symptom and related disorders: A systematic review. J Psychosom Res. 2021;140:110290. 33227556 10.1016/j.jpsychores.2020.110290 PMC7945369

[15] Fang J, Tang H, Liao H, Zhong Y, Li Y, Liao Y, Li Y. Network analysis of Anxiety, Insomnia, Depression, and suicide attempts in Chinese outpatients with somatic symptom disorder. Neuropsych Dis Treat. 2025;21:1091–105. 10.2147/NDT.S512848 PMC12105630 40420960

[16] Ji Y, Liu Z, Jia C, Liu X. Associations between somatic symptoms and suicidal behavior: a cohort study in Chinese adolescents. BMC Psychol. 2025;13(1):788. 40665454 10.1186/s40359-025-03113-0 PMC12261720

[17] Chen J, Lin Y, Hu R, Hu C. Prediction model for depression risk in middle-aged and elderly patients with metabolic syndrome: a nomogram and interpretable machine learning approach based on CHARLS. BMC Psychiatry. 2025;25(1):987. 41088036 10.1186/s12888-025-07434-7 PMC12522473

[18] Cheng X, Liu F, Zhang X, Liu Y, Guo J, Zhong X, Tian D, Pei A, Xiang X, Yao Y, et al. Investigating the role of depression in obstructive sleep apnea and predicting risk factors for OSA in depressed patients: machine learning-assisted evidence from NHANES. BMC Psychiatry. 2025;25(1):964. 41074066 10.1186/s12888-025-07414-x PMC12512373

[19] Ahsan MM, Luna SA, Siddique Z. Machine-learning-based disease diagnosis: a comprehensive review. Healthcare (Basel). 2022;10(3). 10.3390/healthcare10030541 PMC8950225 35327018

[20] Battineni G, Sagaro GG, Chinatalapudi N, Amenta F. Applications of machine learning predictive models in the chronic disease diagnosis. J Pers Med. 2020;10(2). 10.3390/jpm10020021 PMC7354442 32244292

[21] Ge F, Jiang J, Wang Y, Yuan C, Zhang W. Identifying suicidal ideation among Chinese patients with major depressive disorder: evidence from a Real-World Hospital-Based study in China. Neuropsych Dis Treat. 2020;16:665–72. 10.2147/NDT.S238286 PMC7061409 32184605

[22] Hettige NC, Nguyen TB, Yuan C, Rajakulendran T, Baddour J, Bhagwat N, Bani-Fatemi A, Voineskos AN, Mallar Chakravarty M, De Luca V. Classification of suicide attempters in schizophrenia using Sociocultural and clinical features: A machine learning approach. Gen Hosp Psychiat. 2017;47:20–8. 10.1016/j.genhosppsych.2017.03.001 28807134

[23] Gan Y, Kuang L, Xu X, Ai M, He J, Wang W, Hong S, Chen JM, Cao J, Zhang Q. Research on prediction model of adolescent suicide and self-injury behavior based on machine learning algorithm. Front Psychiatry. 2025;15:1521025. 40115313 10.3389/fpsyt.2024.1521025 PMC11922950

[24] Visoki E, Moore TM, Ruiz VM, Fein JA, Calkins ME, Gur RC, Benton TD, Gur RE, Tsui FR, Barzilay R. Prediction of adolescent suicide attempt by integrating Clinical, neurocognitive and geocoded neighborhood environment data. Schizophrenia Bull. 2025;51(4):895–905. 10.1093/schbul/sbaf064 PMC12236345 40476927

[25] Pigoni A, Delvecchio G, Turtulici N, Madonna D, Pietrini P, Cecchetti L, Brambilla P. Machine learning and the prediction of suicide in psychiatric populations: a systematic review. Transl Psychiat. 2024;14(1):140. 10.1038/s41398-024-02852-9 PMC10925059 38461283

[26] Jadhakhan F, Romeu D, Lindner O, Blakemore A, Guthrie E. Prevalence of medically unexplained symptoms in adults who are high users of healthcare services and magnitude of associated costs: a systematic review. BMJ Open. 2022;12(10):e059971. 36198445 10.1136/bmjopen-2021-059971 PMC9535167

[27] Kroenke K. Patients presenting with somatic complaints: epidemiology, psychiatric comorbidity and management. Int J Meth Psych Res. 2003;12(1):34–43. 10.1002/mpr.140 PMC6878426 12830308

[28] Husain M, Chalder T. Medically unexplained symptoms: assessment and management. Clinical medicine. (London England). 2021;21(1):13–8. 10.7861/clinmed.2020-0947 PMC7850206 33479063

[29] Huang W, Chiu Y, Liao S, Wu C. Neuropsychological features of somatic symptom disorder and depression/anxiety in taiwan: an analysis based on the comorbidity status. Psychiat Res. 2024;340:116103. 10.1016/j.psychres.2024.116103 39106815

[30] Ponce-Bobadilla AV, Schmitt V, Maier CS, Mensing S, Stodtmann S. Practical guide to SHAP analysis: explaining supervised machine learning model predictions in drug development. CTS-Clin Transl Sci. 2024;17(11):e70056. 10.1111/cts.70056 PMC11513550 39463176

[31] Eddington HS, Trickey AW, Shah V, Harris AHS. Tutorial: implementing and visualizing machine learning (ML) clinical prediction models into web-accessible calculators using Shiny R. Ann Transl Med. 2022;10(24):1414. 36660686 10.21037/atm-22-847 PMC9843315

[32] Fang J, Tang H, Liao H, Zhong Y, Li Y, Liao Y, Li Y. Network analysis of Anxiety, Insomnia, Depression, and suicide attempts in Chinese outpatients with somatic symptom disorder. Neuropsych Dis Treat. 2025;21:1091–105. 10.2147/NDT.S512848 PMC12105630 40420960

[33] Zhan D, Jin T, Zhao H, Zhu H, Fu J, Zhang X, Sun W, Zhang X, Chen P. Hypothalamic-pituitary-thyroid axis dysregulation in adolescents with major depressive disorder and non-suicidal self-injury: A retrospective study. J Affect Disorders. 2026;399:121120. 41485512 10.1016/j.jad.2025.121120

[34] Song X, Yi L, Wang S, Zhang X. Thyroid dysfunction in elderly first-episode drug-naïve major depressive disorder patients with comorbid dyslipidemia: prevalence, clinical profile and associated factors. Front Psychiatry. 2026;16:1742901. 41601507 10.3389/fpsyt.2025.1742901 PMC12834124

[35] Hu J, Ji Y, Lang X, Zhang X. Association of thyroid function with abnormal lipid metabolism in young patients with first-episode and drug naïve major depressive disorder. Front Psychiatry. 2023;14:1085105. 36865071 10.3389/fpsyt.2023.1085105 PMC9971224

[36] HAMILTON M. A rating scale for depression. J Neurol Neurosurg Psychiatry. 1960;23(1):56–62. 14399272 10.1136/jnnp.23.1.56 PMC495331

[37] HAMILTON M. The assessment of anxiety States by rating. Br J Med Psychol. 1959;32(1):50–5. 13638508 10.1111/j.2044-8341.1959.tb00467.x

[38] Kroenke K, Spitzer RL, Williams JB. The PHQ-9: validity of a brief depression severity measure. J Gen Intern Med. 2001;16(9):606–13. 11556941 10.1046/j.1525-1497.2001.016009606.x PMC1495268

[39] Swinson RP. The GAD-7 scale was accurate for diagnosing generalised anxiety disorder. Evid Based Med. 2006;11(6):184. 17213178 10.1136/ebm.11.6.184

[40] Spitzer RL, Kroenke K, Williams JBW, Löwe B. A brief measure for assessing generalized anxiety disorder: the GAD-7. Arch Intern Med. 2006;166(10):1092–7. 16717171 10.1001/archinte.166.10.1092

[41] Bastien CH, Vallières A, Morin CM. Validation of the insomnia severity index as an outcome measure for insomnia research. Sleep Med. 2001;2(4):297–307. 11438246 10.1016/s1389-9457(00)00065-4

[42] Rosenberg M. Society and the adolescent self-image, rev. ed. Society and the adolescent self-image, rev. ed. Middletown, CT, England: Wesleyan University; 1989. p. 347.

[43] Hays RD, DiMatteo MR. A short-form measure of loneliness. J Pers Assess. 1987;51(1):69–81. 3572711 10.1207/s15327752jpa5101_6

[44] Snyder CR, Harris C, Anderson JR, Holleran SA, Irving LM, Sigmon ST, Yoshinobu L, Gibb J, Langelle C, Harney P. The will and the ways: development and validation of an individual-differences measure of hope. J Pers Soc Psychol. 1991;60(4):570–85. 2037968 10.1037//0022-3514.60.4.570

[45] Schwarzer R, Jerusalem M. Generalized self-efficacy scale. In: Weinman J, Wright S, Johnston M, editors. Windsor (UK): NFER-NELSON; 1995. p. 35–37.

[46] Cohen S, Kamarck T, Mermelstein R. A global measure of perceived stress. J Health Soc Behav. 1983;24(4):385–96. 6668417

[47] Campbell-Sills L, Stein MB. Psychometric analysis and refinement of the Connor–Davidson resilience scale (CD-RISC): validation of a 10-item measure of resilience. J Trauma Stress. 2007;20(6):1019–28. 18157881 10.1002/jts.20271

[48] Bagby RM, Parker JD, Taylor GJ. The twenty-item Toronto alexithymia Scale–I. Item selection and cross-validation of the factor structure. J Psychosom Res. 1994;38(1):23–32. 8126686 10.1016/0022-3999(94)90005-1

[49] Treynor W, Gonzalez R, Nolen-Hoeksema S. Rumination reconsidered: A psychometric analysis. Cogn Ther Res. 2003;27(3):247–59.

[50] Baer RA, Smith GT, Hopkins J, Krietemeyer J, Toney L. Using self-report assessment methods to explore facets of mindfulness. Assessment. 2006;13(1):27–45. 16443717 10.1177/1073191105283504

[51] Diener E, Emmons RA, Larsen RJ, Griffin S. The satisfaction with life scale. J Pers Assess. 1985;49(1):71–5. 16367493 10.1207/s15327752jpa4901_13

[52] Neff KD. Development and validation of a scale to measure self-compassion. Self Identity. 2003;2(3):223–50.

[53] Bond FW, Hayes SC, Baer RA, Carpenter KM, Guenole N, Orcutt HK, Waltz T, Zettle RD. Preliminary psychometric properties of the acceptance and action Questionnaire-II: A revised measure of psychological inflexibility and experiential avoidance. Behav Ther. 2011;42(4):676–88. 22035996 10.1016/j.beth.2011.03.007

[54] Zimet GD, Dahlem NW, Zimet SG, Farley GK. The multidimensional scale of perceived social support. J Pers Assess. 1988;52(1):30–41. 10.1080/00223891.1990.9674095 2280326

[55] Norbeck JS, Lindsey AM, Carrieri VL. The development of an instrument to measure social support. Nurs Res. 1981;30(5):264–9. 7027185

[56] Randolph C, Tierney MC, Mohr E, Chase TN. The repeatable battery for the assessment of neuropsychological status (RBANS): preliminary clinical validity. J Clin Exp Neuropsyc. 1998;20(3):310–9. 10.1076/jcen.20.3.310.823 9845158

[57] Kay SR, Fiszbein A, Opler LA. The positive and negative syndrome scale (PANSS) for schizophrenia. Schizophrenia Bull. 1987;13(2):261–76. 10.1093/schbul/13.2.261 3616518

[58] Guy W, Rockville MD. U.S. Department of Health, Education, and Welfare; National Institute of Mental Health; 1976. p.218–22.

[59] Hu J, Huang Y, Zhang X, Liao B, Hou G, Xu Z, Dong S, Li P. Identifying suicide attempts, ideation, and non-ideation in major depressive disorder from structural MRI data using deep learning. Asian J Psychiatr. 2023;82:103511. 36791609 10.1016/j.ajp.2023.103511

[60] Kang L, Wang W, Zhang N, Nie Z, Gong Q, Yao L, Tu N, Feng H, Zong X, Bai H, et al. Superior Temporal gyrus and cerebellar loops predict nonsuicidal self-injury in major depressive disorder patients by multimodal neuroimaging. Transl Psychiat. 2022;12(1):474. 10.1038/s41398-022-02235-y PMC9649804 36357369

[61] Bhak Y, Jeong H, Cho YS, Jeon S, Cho J, Gim J, Jeon Y, Blazyte A, Park SG, Kim H, et al. Depression and suicide risk prediction models using blood-derived multi-omics data. Transl Psychiat. 2019;9(1):262. 10.1038/s41398-019-0595-2 PMC6797735 31624227

[62] Saeed S, Wang H, Kong L, Geng Y, Zhang J, Pan Y, Le X, Zhang X, Liu TT, Hu S. Machine learning-enhanced mapping of suicide risk in bipolar disorder: A multi-modal analysis. J Affect Disorders. 2026;392:120183. 40921216 10.1016/j.jad.2025.120183

[63] Palagini L, Geoffroy PA, Miniati M, Riemann D, Gemignani A, Marazziti D. Insomnia and circadian rhythms dysregulation in people who have attempted suicide: correlations with markers of inflammation and suicidal lethality. World J Biol Psychia. 2024;25(7):408–16. 10.1080/15622975.2024.2391456 39163256

[64] Sun H, Chen P, Bai W, Zhang L, Feng Y, Su Z, Cheung T, Ungvari GS, Cui X, Ng CH, et al. Prevalence and network structure of depression, insomnia and suicidality among mental health professionals who recovered from COVID-19: a National survey in China. Transl Psychiat. 2024;14(1):227. 10.1038/s41398-024-02918-8 PMC11139988 38816419

[65] Yi P, Qin Y, Zheng C, Ren K, Huang L, Chen W. Tumor markers and depression scores are predictive of non-suicidal self-injury behaviors among adolescents with depressive disorder: A retrospective study. Front Neurosci-Switz. 2022;16:953842. 10.3389/fnins.2022.953842 PMC9403252 36033621

[66] Wu F, Yi Y, Lian Y, Chen Q, Luo L, Yang H, Li H, Feng Y, Feng S, Zhou S, et al. Sex differences in the association between suicidal ideation and neurocognitive function in Chinese patients with schizophrenia. Eur Arch Psy Clin N. 2024;274(6):1355–63. 10.1007/s00406-023-01616-8 37184751

[67] Lu C, Qi D, Ping Y, Zhang C, Wang S, Liu N, Wang X, Li S, Li J. Suicide risk, psychopathology and cognitive impairments in schizophrenia with insomnia: a large-scale cross-sectional study. BMC Psychiatry. 2025;25(1):920. 41039393 10.1186/s12888-025-07306-0 PMC12490073

[68] Yang W, Wang X, Kang C, Yang L, Liu D, Zhao N, Zhang X. Establishment of a risk prediction model for suicide attempts in first-episode and drug Naive patients with major depressive disorder. Asian J Psychiatr. 2023;88:103732. 37586124 10.1016/j.ajp.2023.103732

[69] Liu W, Wu Z, Sun M, Zhang S, Yuan J, Zhu D, Yan G, Hou K. Association between fasting blood glucose and thyroid stimulating hormones and suicidal tendency and disease severity in patients with major depressive disorder. Bosnian J Basic Med. 2022;22(4):635–42. 10.17305/bjbms.2021.6754 PMC9392982 35238287

[70] Lee J, Lee S, Kim M, Kwon H, Yun J, Yang Y, Yoon K, Cho J, Pae C, Han K, et al. Inverse association between obesity and suicidal death risk. BMC Psychiatry. 2025;25(1):27. 39780112 10.1186/s12888-024-06381-z PMC11714859

