# Supplementary material for: Identifying past-year self-reported suicidality in outpatients with somatic symptom disorder using an interpretable machine-learning model: a multicenter study with an online calculator
Source: BMC Psychiatry. 2026 Feb 18;26:255. doi: 10.1186/s12888-026-07901-9 (PMC13020323; doi:10.1186/s12888-026-07901-9)
Supplement: Supplementary file 1 — Supplementary Material 1 [file 12888_2026_7901_MOESM1_ESM.docx]

Table S1. Prespecified interaction terms tested in logistic regression

| Interaction | β (SE) | OR (95% CI) | P value |
| --- | --- | --- | --- |
| Psychotic symptoms × PHQ-9 | 0.3443 (0.1032) | 1.411 (1.153–1.727) | <0.001 |
| PHQ-9 × ISI | 0.0155 (0.0056) | 1.016 (1.005–1.027) | 0.006 |
| PHQ-9 × GAD-7 | -0.0146 (0.0079) | 0.985 (0.970–1.001) | 0.064 |
| ULS-8 × PSSS | -0.0045 (0.0026) | 0.996 (0.990–1.001) | 0.084 |
| Sex × PHQ-9 | 0.0015 (0.0346) | 1.001 (0.936–1.072) | 0.966 |

Interaction terms were prespecified based on clinical plausibility and tested in a logistic regression model. Values are reported as β (SE) and OR (95% CI), where OR represents the multiplicative change in odds associated with a one-unit increase in one predictor conditional on the level of the other predictor. Psychotic symptoms was coded as a binary variable (Yes/No). P values are two-sided. OR, odds ratio; CI, confidence interval; SE, standard error; ISI, Insomnia Severity Index; PHQ-9, Patient Health Questionnaire-9; GAD-7, Generalized Anxiety Disorder-7; ULS-8, 8-item UCLA Loneliness Scale; PSSS, Perceived Social Support Scale.
