# Supplementary material for: Identifying past-year self-reported suicidality in outpatients with somatic symptom disorder using an interpretable machine-learning model: a multicenter study with an online calculator
Source: BMC Psychiatry. 2026 Feb 18;26:255. doi: 10.1186/s12888-026-07901-9 (PMC13020323; doi:10.1186/s12888-026-07901-9)
Supplement: Supplementary file 2 — Supplementary Material 2 [file 12888_2026_7901_MOESM2_ESM.docx]

Table S2. Model performance with vs without prespecified interaction terms

| Model | AUROC | Brier score | Calibration intercept | Calibration slope |
| --- | --- | --- | --- | --- |
| Main-effects model | 0.910 | 0.085 | 0.000 | 1.000 |
| Main-effects + interactions | 0.920 | 0.081 | -0.000 | 1.000 |

AUROC indicates discrimination performance. Brier score reflects overall accuracy of predicted probabilities. Calibration intercept and slope were estimated by regressing the observed outcome on the logit of predicted probabilities; an intercept close to 0 and slope close to 1 indicate good calibration. AUROC, area under the receiver operating characteristic curve.
