# Supplementary material for: Identifying past-year self-reported suicidality in outpatients with somatic symptom disorder using an interpretable machine-learning model: a multicenter study with an online calculator
Source: BMC Psychiatry. 2026 Feb 18;26:255. doi: 10.1186/s12888-026-07901-9 (PMC13020323; doi:10.1186/s12888-026-07901-9)
Supplement: Supplementary file 3 — Supplementary Material 3 [file 12888_2026_7901_MOESM3_ESM.docx]

Table S3. Model performance across 10 repeated random train–test splits using different random seeds

| Seed | Model | AUC | | AUPRC | |
| --- | --- | --- | --- | --- | --- |
|  |  | Train | Test | Train | Test |
| 190000 | GBM | 0.999 (0.998–1.000) | 0.936 (0.886–0.986) | 0.997 | 0.909 |
|  | GLM | 0.971 (0.957–0.986) | 0.930 (0.880–0.979) | 0.895 | 0.884 |
|  | GLMNET | 0.971 (0.956–0.986) | 0.931 (0.882–0.980) | 0.896 | 0.884 |
|  | RANGER | 1.000 (1.000–1.000) | 0.954 (0.912–0.996) | 1.000 | 0.928 |
|  | SVM | 0.985 (0.975–0.995) | 0.943 (0.899–0.986) | 0.938 | 0.893 |
|  | XGB | 0.986 (0.976–0.996) | 0.928 (0.879–0.976) | 0.949 | 0.890 |
|  | NB | 0.979 (0.969–0.990) | 0.913 (0.857–0.969) | 0.929 | 0.838 |
|  | NNET | 0.978 (0.968–0.988) | 0.942 (0.894–0.990) | 0.889 | 0.861 |
| 1994061212 | GBM | 0.970 (0.950–0.989) | 0.973 (0.945–1.000) | 0.924 | 0.954 |
|  | GLM | 0.959 (0.937–0.980) | 0.973 (0.951–0.995) | 0.881 | 0.931 |
|  | GLMNET | 0.956 (0.934–0.979) | 0.969 (0.944–0.995) | 0.881 | 0.933 |
|  | RANGER | 1.000 (0.999–1.000) | 0.978 (0.952–1.000) | 0.999 | 0.970 |
|  | SVM | 0.987 (0.979–0.996) | 0.974 (0.948–1.000) | 0.961 | 0.948 |
|  | XGB | 0.976 (0.959–0.993) | 0.969 (0.935–1.000) | 0.932 | 0.941 |
|  | NB | 0.953 (0.934–0.972) | 0.948 (0.921–0.975) | 0.860 | 0.851 |
|  | NNET | 0.961 (0.938–0.985) | 0.945 (0.909–0.980) | 0.891 | 0.816 |
| 19940612 | GBM | 0.994 (0.990–0.998) | 0.964 (0.929–0.998) | 0.978 | 0.945 |
|  | GLM | 0.963 (0.944–0.981) | 0.963 (0.929–0.997) | 0.881 | 0.932 |
|  | GLMNET | 0.962 (0.943–0.981) | 0.963 (0.929–0.997) | 0.880 | 0.936 |
|  | RANGER | 0.999 (0.998–1.000) | 0.978 (0.955–1.000) | 0.997 | 0.960 |
|  | SVM | 0.989 (0.980–0.997) | 0.964 (0.932–0.996) | 0.970 | 0.937 |
|  | XGB | 0.982 (0.970–0.994) | 0.966 (0.933–1.000) | 0.942 | 0.948 |
|  | NB | 0.964 (0.949–0.980) | 0.919 (0.877–0.961) | 0.890 | 0.709 |
|  | NNET | 0.999 (0.998–1.000) | 0.962 (0.926–0.997) | 0.998 | 0.934 |
| 2000000 | GBM | 0.999 (0.998–1.000) | 0.969 (0.941–0.996) | 0.996 | 0.932 |
|  | GLM | 0.960 (0.941–0.978) | 0.972 (0.944–1.000) | 0.883 | 0.993 |
|  | GLMNET | 0.959 (0.940–0.978) | 0.973 (0.945–1.000) | 0.884 | 0.933 |
|  | RANGER | 1.000 (1.000–1.000) | 0.989 (0.975–1.000) | 1.000 | 0.967 |
|  | SVM | 0.990 (0.983–0.997) | 0.981 (0.955–1.000) | 0.959 | 0.962 |
|  | XGB | 0.989 (0.981–0.997) | 0.974 (0.943–1.000) | 0.965 | 0.954 |
|  | NB | 0.968 (0.952–0.984) | 0.921 (0.887–0.955) | 0.918 | 0.627 |
|  | NNET | 0.971 (0.957–0.984) | 0.967 (0.926–1.000) | 0.873 | 0.942 |
| 13131313 | GBM | 0.999 (0.998–1.000) | 0.987 (0.972–1.000) | 0.995 | 0.981 |
|  | GLM | 0.956 (0.934–0.978) | 0.977 (0.957–0.996) | 0.859 | 0.948 |
|  | GLMNET | 0.955 (0.933–0.977) | 0.977 (0.957–0.997) | 0.861 | 0.952 |
|  | RANGER | 1.000 (1.000–1.000) | 0.986 (0.972–1.000) | 0.999 | 0.976 |
|  | SVM | 0.984 (0.972–0.995) | 0.985 (0.972–0.998) | 0.935 | 0.966 |
|  | XGB | 0.971 (0.953–0.989) | 0.984 (0.965–1.000) | 0.922 | 0.978 |
|  | NB | 0.960 (0.942–0.979) | 0.939 (0.909–0.969) | 0.873 | 0.779 |
|  | NNET | 0.972 (0.959–0.985) | 0.954 (0.921–0.988) | 0.874 | 0.926 |
| 555555 | GBM | 1.000 (1.000–1.000) | 0.969 (0.937–1.000) | 1.000 | 0.955 |
|  | GLM | 0.966 (0.949–0.983) | 0.964 (0.934–0.994) | 0.877 | 0.929 |
|  | GLMNET | 0.964 (0.946–0.982) | 0.964 (0.934–0.994) | 0.878 | 0.931 |
|  | RANGER | 1.000 (1.000–1.000) | 0.973 (0.941–1.000) | 0.999 | 0.962 |
|  | SVM | 0.988 (0.979–0.997) | 0.974 (0.952–0.997) | 0.956 | 0.938 |
|  | XGB | 0.981 (0.968–0.994) | 0.969 (0.940–0.998) | 0.939 | 0.947 |
|  | NB | 0.965 (0.948–0.981) | 0.940 (0.905–0.975) | 0.896 | 0.795 |
|  | NNET | 0.978 (0.965–0.990) | 0.956 (0.920–0.992) | 0.888 | 0.889 |
| 20260119 | GBM | 1.000 (1.000–1.000) | 0.952 (0.912–0.993) | 1.000 | 0.898 |
|  | GLM | 0.974 (0.960–0.988) | 0.940 (0.899–0.981) | 0.912 | 0.860 |
|  | GLMNET | 0.973 (0.957–0.988) | 0.940 (0.898–0.981) | 0.911 | 0.867 |
|  | RANGER | 1.000 (1.000–1.000) | 0.963 (0.928–0.998) | 1.000 | 0.929 |
|  | SVM | 0.985 (0.974–0.996) | 0.955 (0.924–0.986) | 0.949 | 0.848 |
|  | XGB | 0.989 (0.981–0.997) | 0.957 (0.926–0.988) | 0.964 | 0.897 |
|  | NB | 0.933 (0.911–0.954) | 0.894 (0.844–0.944) | 0.744 | 0.654 |
|  | NNET | 0.985 (0.976–0.994) | 0.945 (0.909–0.982) | 0.936 | 0.819 |
| 007 | GBM | 0.994 (0.990–0.998) | 0.978 (0.957–1.000) | 0.978 | 0.958 |
|  | GLM | 0.961 (0.940–0.982) | 0.967 (0.942–0.993) | 0.891 | 0.922 |
|  | GLMNET | 0.958 (0.936–0.980) | 0.960 (0.929–0.990) | 0.888 | 0.915 |
|  | RANGER | 0.999 (0.999–1.000) | 0.972 (0.942–1.000) | 0.997 | 0.953 |
|  | SVM | 0.989 (0.980–0.999) | 0.970 (0.947–0.992) | 0.975 | 0.922 |
|  | XGB | 0.983 (0.972–0.994) | 0.969 (0.940–0.997) | 0.938 | 0.948 |
|  | NB | 0.965 (0.951–0.980) | 0.931 (0.892–0.971) | 0.891 | 0.816 |
|  | NNET | 0.962 (0.941–0.983) | 0.953 (0.918–0.989) | 0.874 | 0.886 |
| 10086 | GBM | 0.986 (0.977–0.996) | 0.968 (0.939–0.996) | 0.963 | 0.900 |
|  | GLM | 0.962 (0.942–0.983) | 0.962 (0.935–0.988) | 0.904 | 0.870 |
|  | GLMNET | 0.962 (0.941–0.983) | 0.961 (0.934–0.988) | 0.904 | 0.872 |
|  | RANGER | 1.000 (1.000–1.000) | 0.986 (0.973–1.000) | 1.000 | 0.937 |
|  | SVM | 0.986 (0.975–0.996) | 0.971 (0.948–0.993) | 0.965 | 0.868 |
|  | XGB | 0.978 (0.964–0.992) | 0.956 (0.919–0.993) | 0.940 | 0.911 |
|  | NB | 0.976 (0.964–0.988) | 0.907 (0.845–0.968) | 0.926 | 0.762 |
|  | NNET | 0.978 (0.966–0.989) | 0.921 (0.869–0.972) | 0.930 | 0.804 |
| 520 | GBM | 0.999 (0.998–1.000) | 0.999 (0.998–1.000) | 0.997 | 0.899 |
|  | GLM | 0.968 (0.952–0.984) | 0.968 (0.952–0.984) | 0.904 | 0.867 |
|  | GLMNET | 0.968 (0.952–0.984) | 0.968 (0.952–0.984) | 0.905 | 0.868 |
|  | RANGER | 1.000 (1.000–1.000) | 1.000 (1.000–1.000) | 1.000 | 0.942 |
|  | SVM | 0.992 (0.987–0.998) | 0.992 (0.987–0.998) | 0.972 | 0.800 |
|  | XGB | 0.995 (0.992–0.998) | 0.995 (0.992–0.998) | 0.984 | 0.892 |
|  | NB | 0.960 (0.945–0.976) | 0.960 (0.945–0.976) | 0.885 | 0.808 |
|  | NNET | 0.985 (0.977–0.993) | 0.985 (0.977–0.993) | 0.928 | 0.703 |

AUC, area under the receiver operating characteristic curve; AUPRC, area under the precision–recall curve; GBM, gradient boosting machine; GLM, generalized linear model (logistic regression); GLMNET, penalized logistic regression (elastic net); RANGER, random forest; SVM, support vector machine; XGB, extreme gradient boosting; NB, naïve Bayes; NNET, neural network.
