# Supplementary material for: Identifying past-year self-reported suicidality in outpatients with somatic symptom disorder using an interpretable machine-learning model: a multicenter study with an online calculator
Source: BMC Psychiatry. 2026 Feb 18;26:255. doi: 10.1186/s12888-026-07901-9 (PMC13020323; doi:10.1186/s12888-026-07901-9)
Supplement: Supplementary file 4 — Supplementary Material 4 [file 12888_2026_7901_MOESM4_ESM.docx]

Table S4. Discrimination performance of the questionnaire-only model across 10 repeated train–test splits

| Seed | Model | AUC | | AUPRC | |
| --- | --- | --- | --- | --- | --- |
|  |  | Train | Test | Train | Test |
| 190000 | GBM | 0.997 (0.994–1.000) | 0.915 (0.858–0.973) | 0.988 | 0.804 |
|  | GLM | 0.965 (0.947–0.984) | 0.914 (0.858–0.970) | 0.868 | 0.860 |
|  | GLMNET | 0.964 (0.945–0.983) | 0.914 (0.858–0.970) | 0.866 | 0.860 |
|  | RANGER | 0.997 (0.995–1.000) | 0.915 (0.855–0.974) | 0.991 | 0.890 |
|  | SVM | 0.991 (0.984–0.997) | 0.906 (0.847–0.965) | 0.965 | 0.870 |
|  | XGB | 0.978 (0.962–0.993) | 0.925 (0.875–0.974) | 0.923 | 0.887 |
|  | NB | 0.979 (0.967–0.991) | 0.918 (0.862–0.973) | 0.942 | 0.880 |
|  | NNET | 0.989 (0.981–0.998) | 0.907 (0.842–0.973) | 0.976 | 0.882 |
| 1994061212 | GBM | 0.994 (0.989–0.998) | 0.943 (0.898–0.988) | 0.980 | 0.894 |
|  | GLM | 0.953 (0.930–0.975) | 0.940 (0.891–0.989) | 0.862 | 0.878 |
|  | GLMNET | 0.952 (0.929–0.975) | 0.940 (0.890–0.989) | 0.865 | 0.852 |
|  | RANGER | 0.995 (0.990–0.999) | 0.959 (0.919–0.998) | 0.983 | 0.936 |
|  | SVM | 0.987 (0.978–0.996) | 0.949 (0.907–0.990) | 0.967 | 0.888 |
|  | XGB | 0.977 (0.961–0.993) | 0.932 (0.880–0.983) | 0.952 | 0.852 |
|  | NB | 0.964 (0.946–0.981) | 0.948 (0.909–0.988) | 0.907 | 0.884 |
|  | NNET | 0.972 (0.955–0.989) | 0.914 (0.850–0.978) | 0.917 | 0.781 |
| 19940612 | GBM | 0.960 (0.936–0.983) | 0.957 (0.919–0.995) | 0.882 | 0.937 |
|  | GLM | 0.947 (0.922–0.973) | 0.955 (0.917–0.993) | 0.843 | 0.921 |
|  | GLMNET | 0.947 (0.920–0.973) | 0.955 (0.917–0.994) | 0.842 | 0.921 |
|  | RANGER | 0.989 (0.981–0.997) | 0.971 (0.939–1.000) | 0.971 | 0.956 |
|  | SVM | 0.984 (0.974–0.995) | 0.960 (0.922–0.998) | 0.951 | 0.944 |
|  | XGB | 0.961 (0.939–0.983) | 0.959 (0.924–0.995) | 0.915 | 0.940 |
|  | NB | 0.960 (0.942–0.979) | 0.954 (0.917–0.990) | 0.889 | 0.916 |
|  | NNET | 0.972 (0.958–0.986) | 0.949 (0.909–0.989) | 0.908 | 0.894 |
| 2000000 | GBM | 0.988 (0.979–0.997) | 0.972 (0.944–1.000) | 0.970 | 0.944 |
|  | GLM | 0.936 (0.908–0.964) | 0.968 (0.933–1.000) | 0.829 | 0.947 |
|  | GLMNET | 0.936 (0.908–0.964) | 0.969 (0.934–1.000) | 0.829 | 0.947 |
|  | RANGER | 0.993 (0.988–0.998) | 0.978 (0.951–1.000) | 0.980 | 0.957 |
|  | SVM | 0.979 (0.966–0.991) | 0.960 (0.928–0.993) | 0.941 | 0.918 |
|  | XGB | 0.965 (0.945–0.984) | 0.971 (0.937–1.000) | 0.928 | 0.956 |
|  | NB | 0.967 (0.949–0.984) | 0.960 (0.927–0.993) | 0.925 | 0.913 |
|  | NNET | 0.986 (0.978–0.994) | 0.974 (0.950–0.999) | 0.954 | 0.944 |
| 13131313 | GBM | 0.993 (0.988–0.998) | 0.974 (0.953–0.994) | 0.972 | 0.957 |
|  | GLM | 0.935 (0.903–0.967) | 0.969 (0.943–0.995) | 0.809 | 0.947 |
|  | GLMNET | 0.934 (0.902–0.966) | 0.968 (0.942–0.994) | 0.813 | 0.945 |
|  | RANGER | 0.993 (0.987–0.998) | 0.972 (0.945–1.000) | 0.971 | 0.967 |
|  | SVM | 0.974 (0.961–0.986) | 0.975 (0.952–0.997) | 0.887 | 0.960 |
|  | XGB | 0.964 (0.942–0.985) | 0.975 (0.952–0.998) | 0.915 | 0.966 |
|  | NB | 0.963 (0.943–0.983) | 0.972 (0.952–0.992) | 0.901 | 0.953 |
|  | NNET | 0.958 (0.940–0.977) | 0.977 (0.958–0.996) | 0.805 | 0.940 |
| 555555 | GBM | 0.991 (0.984–0.997) | 0.955 (0.918–0.992) | 0.971 | 0.933 |
|  | GLM | 0.950 (0.925–0.975) | 0.948 (0.910–0.986) | 0.845 | 0.900 |
|  | GLMNET | 0.949 (0.923–0.975) | 0.949 (0.911–0.987) | 0.844 | 0.903 |
|  | RANGER | 0.995 (0.991–0.999) | 0.959 (0.922–0.996) | 0.984 | 0.943 |
|  | SVM | 0.969 (0.953–0.985) | 0.954 (0.925–0.983) | 0.874 | 0.858 |
|  | XGB | 0.975 (0.959–0.991) | 0.955 (0.918–0.993) | 0.933 | 0.939 |
|  | NB | 0.970 (0.953–0.986) | 0.948 (0.909–0.988) | 0.923 | 0.930 |
|  | NNET | 0.980 (0.965–0.994) | 0.949 (0.909–0.989) | 0.955 | 0.921 |
| 20260119 | GBM | 0.995 (0.991–0.999) | 0.951 (0.908–0.993) | 0.982 | 0.923 |
|  | GLM | 0.962 (0.942–0.983) | 0.918 (0.868–0.969) | 0.881 | 0.818 |
|  | GLMNET | 0.959 (0.937–0.981) | 0.919 (0.867–0.970) | 0.878 | 0.832 |
|  | RANGER | 0.996 (0.993–0.999) | 0.942 (0.893–0.991) | 0.987 | 0.900 |
|  | SVM | 0.987 (0.979–0.994) | 0.932 (0.890–0.975) | 0.944 | 0.837 |
|  | XGB | 0.971 (0.952–0.990) | 0.944 (0.904–0.984) | 0.923 | 0.891 |
|  | NB | 0.976 (0.961–0.990) | 0.936 (0.888–0.983) | 0.943 | 0.892 |
|  | NNET | 0.973 (0.953–0.994) | 0.927 (0.872–0.981) | 0.955 | 0.900 |
| 007 | GBM | 0.994 (0.989–0.999) | 0.948 (0.903–0.992) | 0.985 | 0.870 |
|  | GLM | 0.952 (0.926–0.978) | 0.940 (0.901–0.980) | 0.880 | 0.839 |
|  | GLMNET | 0.951 (0.924–0.977) | 0.939 (0.898–0.980) | 0.880 | 0.840 |
|  | RANGER | 0.996 (0.993–0.999) | 0.951 (0.903–0.998) | 0.988 | 0.893 |
|  | SVM | 0.990 (0.983–0.997) | 0.944 (0.894–0.994) | 0.966 | 0.914 |
|  | XGB | 0.967 (0.948–0.986) | 0.944 (0.901–0.987) | 0.932 | 0.890 |
|  | NB | 0.970 (0.955–0.985) | 0.913 (0.853–0.973) | 0.928 | 0.814 |
|  | NNET | 0.984 (0.971–0.997) | 0.935 (0.880–0.990) | 0.969 | 0.874 |
| 10086 | GBM | 0.974 (0.955–0.994) | 0.961 (0.927–0.996) | 0.925 | 0.941 |
|  | GLM | 0.946 (0.918–0.974) | 0.952 (0.917–0.987) | 0.854 | 0.892 |
|  | GLMNET | 0.945 (0.917–0.974) | 0.950 (0.914–0.987) | 0.856 | 0.894 |
|  | RANGER | 0.991 (0.984–0.998) | 0.970 (0.939–1.000) | 0.973 | 0.953 |
|  | SVM | 0.973 (0.958–0.988) | 0.956 (0.922–0.989) | 0.903 | 0.914 |
|  | XGB | 0.961 (0.938–0.983) | 0.955 (0.919–0.990) | 0.915 | 0.926 |
|  | NB | 0.963 (0.945–0.981) | 0.951 (0.916–0.986) | 0.891 | 0.913 |
|  | NNET | 0.980 (0.966–0.994) | 0.978 (0.961–0.995) | 0.945 | 0.929 |
| 520 | GBM | 0.978 (0.964–0.993) | 0.955 (0.904–1.000) | 0.947 | 0.929 |
|  | GLM | 0.947 (0.922–0.973) | 0.950 (0.907–0.993) | 0.870 | 0.855 |
|  | GLMNET | 0.946 (0.921–0.972) | 0.951 (0.908–0.993) | 0.869 | 0.860 |
|  | RANGER | 0.993 (0.988–0.998) | 0.970 (0.940–1.000) | 0.980 | 0.922 |
|  | SVM | 0.978 (0.967–0.990) | 0.963 (0.928–0.998) | 0.932 | 0.925 |
|  | XGB | 0.974 (0.959–0.990) | 0.956 (0.906–1.000) | 0.948 | 0.924 |
|  | NB | 0.965 (0.948–0.982) | 0.971 (0.942–0.999) | 0.924 | 0.931 |
|  | NNET | 0.974 (0.961–0.987) | 0.955 (0.917–0.994) | 0.908 | 0.854 |

AUC, area under the receiver operating characteristic curve; AUPRC, area under the precision–recall curve; GBM, gradient boosting machine; GLM, generalized linear model (logistic regression); GLMNET, penalized logistic regression (elastic net); RANGER, random forest; SVM, support vector machine; XGB, extreme gradient boosting; NB, naïve Bayes; NNET, neural network.
