# Supplementary material for: Identifying past-year self-reported suicidality in outpatients with somatic symptom disorder using an interpretable machine-learning model: a multicenter study with an online calculator
Source: BMC Psychiatry. 2026 Feb 18;26:255. doi: 10.1186/s12888-026-07901-9 (PMC13020323; doi:10.1186/s12888-026-07901-9)
Supplement: Supplementary file 5 — Supplementary Material 5 [file 12888_2026_7901_MOESM5_ESM.docx]

Table S5. Discrimination performance of the laboratory and vital-sign–only model across 10 repeated train–test splits

| Seed | Model | AUC | | AUPRC | |
| --- | --- | --- | --- | --- | --- |
|  |  | Train | Test | Train | Test |
| 190000 | GBM | 0.894 (0.864–0.923) | 0.735 (0.652–0.819) | 0.692 | 0.486 |
|  | GLM | 0.764 (0.714–0.814) | 0.725 (0.642–0.809) | 0.510 | 0.501 |
|  | GLMNET | 0.759 (0.710–0.809) | 0.734 (0.651–0.818) | 0.498 | 0.527 |
|  | RANGER | 0.997 (0.995–1.000) | 0.770 (0.688–0.853) | 0.988 | 0.589 |
|  | SVM | 0.835 (0.789–0.882) | 0.756 (0.668–0.843) | 0.715 | 0.616 |
|  | XGB | 0.874 (0.843–0.906) | 0.746 (0.660–0.832) | 0.657 | 0.552 |
|  | NB | 0.752 (0.703–0.800) | 0.755 (0.669–0.841) | 0.426 | 0.537 |
|  | NNET | 0.791 (0.745–0.836) | 0.742 (0.665–0.819) | 0.546 | 0.497 |
| 1994061212 | GBM | 0.903 (0.874–0.932) | 0.765 (0.693–0.837) | 0.704 | 0.581 |
|  | GLM | 0.751 (0.697–0.805) | 0.758 (0.688–0.829) | 0.482 | 0.558 |
|  | GLMNET | 0.747 (0.694–0.801) | 0.756 (0.684–0.827) | 0.476 | 0.558 |
|  | RANGER | 0.996 (0.993–0.999) | 0.776 (0.704–0.847) | 0.980 | 0.643 |
|  | SVM | 0.875 (0.831–0.919) | 0.709 (0.623–0.794) | 0.786 | 0.597 |
|  | XGB | 0.873 (0.839–0.907) | 0.758 (0.682–0.833) | 0.642 | 0.579 |
|  | NB | 0.751 (0.698–0.805) | 0.763 (0.693–0.833) | 0.443 | 0.515 |
|  | NNET | 0.842 (0.805–0.879) | 0.734 (0.661–0.808) | 0.601 | 0.505 |
| 19940612 | GBM | 0.821 (0.779–0.863) | 0.781 (0.708–0.853) | 0.583 | 0.507 |
|  | GLM | 0.741 (0.688–0.795) | 0.778 (0.708–0.848) | 0.510 | 0.530 |
|  | GLMNET | 0.738 (0.685–0.791) | 0.760 (0.684–0.835) | 0.490 | 0.495 |
|  | RANGER | 0.996 (0.993–0.999) | 0.779 (0.709–0.850) | 0.981 | 0.520 |
|  | SVM | 0.909 (0.872–0.946) | 0.696 (0.605–0.788) | 0.854 | 0.455 |
|  | XGB | 0.843 (0.805–0.881) | 0.792 (0.723–0.862) | 0.613 | 0.519 |
|  | NB | 0.755 (0.704–0.807) | 0.743 (0.664–0.822) | 0.461 | 0.539 |
|  | NNET | 0.756 (0.706–0.807) | 0.751 (0.677–0.824) | 0.579 | 0.516 |
| 2000000 | GBM | 0.830 (0.789–0.871) | 0.741 (0.656–0.825) | 0.612 | 0.445 |
|  | GLM | 0.776 (0.728–0.825) | 0.696 (0.610–0.783) | 0.559 | 0.392 |
|  | GLMNET | 0.777 (0.729–0.825) | 0.695 (0.609–0.782) | 0.554 | 0.390 |
|  | RANGER | 0.996 (0.993–0.999) | 0.752 (0.671–0.834) | 0.982 | 0.439 |
|  | SVM | 0.806 (0.756–0.856) | 0.752 (0.672–0.833) | 0.668 | 0.392 |
|  | XGB | 0.860 (0.825–0.895) | 0.753 (0.673–0.832) | 0.663 | 0.449 |
|  | NB | 0.781 (0.733–0.828) | 0.695 (0.604–0.785) | 0.506 | 0.370 |
|  | NNET | 0.792 (0.745–0.839) | 0.664 (0.578–0.750) | 0.575 | 0.359 |
| 13131313 | GBM | 0.909 (0.881–0.936) | 0.752 (0.683–0.821) | 0.699 | 0.559 |
|  | GLM | 0.768 (0.715–0.822) | 0.734 (0.664–0.805) | 0.500 | 0.553 |
|  | GLMNET | 0.768 (0.715–0.820) | 0.731 (0.660–0.803) | 0.487 | 0.550 |
|  | RANGER | 0.997 (0.994–1.000) | 0.761 (0.691–0.831) | 0.984 | 0.616 |
|  | SVM | 0.837 (0.787–0.888) | 0.704 (0.624–0.784) | 0.688 | 0.606 |
|  | XGB | 0.855 (0.818–0.892) | 0.737 (0.667–0.807) | 0.597 | 0.567 |
|  | NB | 0.763 (0.711–0.816) | 0.736 (0.665–0.807) | 0.428 | 0.506 |
|  | NNET | 0.777 (0.728–0.826) | 0.715 (0.645–0.785) | 0.456 | 0.508 |
| 555555 | GBM | 0.936 (0.915–0.957) | 0.754 (0.679–0.829) | 0.782 | 0.527 |
|  | GLM | 0.762 (0.711–0.813) | 0.739 (0.660–0.817) | 0.490 | 0.564 |
|  | GLMNET | 0.761 (0.711–0.812) | 0.738 (0.659–0.817) | 0.483 | 0.560 |
|  | RANGER | 1.000 (1.000–1.000) | 0.741 (0.663–0.820) | 1.000 | 0.573 |
|  | SVM | 0.869 (0.824–0.913) | 0.697 (0.613–0.782) | 0.790 | 0.474 |
|  | XGB | 0.882 (0.850–0.914) | 0.758 (0.685–0.831) | 0.663 | 0.522 |
|  | NB | 0.765 (0.715–0.814) | 0.744 (0.664–0.824) | 0.448 | 0.518 |
|  | NNET | 0.774 (0.720–0.827) | 0.714 (0.634–0.793) | 0.537 | 0.482 |
| 20260119 | GBM | 0.878 (0.847–0.909) | 0.777 (0.708–0.845) | 0.657 | 0.494 |
|  | GLM | 0.763 (0.712–0.814) | 0.729 (0.648–0.809) | 0.531 | 0.483 |
|  | GLMNET | 0.761 (0.711–0.812) | 0.733 (0.653–0.813) | 0.521 | 0.480 |
|  | RANGER | 0.999 (0.998–1.000) | 0.750 (0.673–0.827) | 0.996 | 0.487 |
|  | SVM | 0.873 (0.827–0.919) | 0.694 (0.605–0.784) | 0.808 | 0.456 |
|  | XGB | 0.890 (0.861–0.920) | 0.760 (0.687–0.833) | 0.686 | 0.512 |
|  | NB | 0.760 (0.710–0.811) | 0.753 (0.676–0.830) | 0.463 | 0.464 |
|  | NNET | 0.747 (0.698–0.797) | 0.681 (0.608–0.755) | 0.602 | 0.436 |
| 007 | GBM | 0.864 (0.829–0.898) | 0.786 (0.716–0.856) | 0.671 | 0.433 |
|  | GLM | 0.759 (0.710–0.808) | 0.733 (0.644–0.823) | 0.507 | 0.498 |
|  | GLMNET | 0.758 (0.708–0.807) | 0.749 (0.664–0.834) | 0.510 | 0.513 |
|  | RANGER | 0.997 (0.994–0.999) | 0.796 (0.721–0.872) | 0.986 | 0.524 |
|  | SVM | 0.835 (0.788–0.882) | 0.683 (0.578–0.787) | 0.725 | 0.532 |
|  | XGB | 0.862 (0.827–0.897) | 0.792 (0.720–0.863) | 0.681 | 0.462 |
|  | NB | 0.748 (0.697–0.799) | 0.786 (0.705–0.867) | 0.481 | 0.509 |
|  | NNET | 0.762 (0.714–0.810) | 0.728 (0.645–0.811) | 0.562 | 0.460 |
| 10086 | GBM | 0.830 (0.789–0.872) | 0.750 (0.673–0.828) | 0.591 | 0.458 |
|  | GLM | 0.772 (0.722–0.822) | 0.718 (0.639–0.796) | 0.523 | 0.465 |
|  | GLMNET | 0.772 (0.722–0.822) | 0.718 (0.640–0.797) | 0.522 | 0.463 |
|  | RANGER | 0.996 (0.993–1.000) | 0.765 (0.691–0.838) | 0.983 | 0.522 |
|  | SVM | 0.891 (0.853–0.929) | 0.676 (0.591–0.761) | 0.808 | 0.449 |
|  | XGB | 0.904 (0.876–0.932) | 0.781 (0.714–0.848) | 0.723 | 0.514 |
|  | NB | 0.781 (0.732–0.830) | 0.702 (0.619–0.784) | 0.510 | 0.428 |
|  | NNET | 0.794 (0.746–0.842) | 0.722 (0.644–0.801) | 0.570 | 0.468 |
| 520 | GBM | 0.902 (0.874–0.930) | 0.802 (0.732–0.871) | 0.719 | 0.570 |
|  | GLM | 0.743 (0.692–0.795) | 0.780 (0.702–0.858) | 0.494 | 0.528 |
|  | GLMNET | 0.742 (0.691–0.793) | 0.782 (0.705–0.859) | 0.489 | 0.531 |
|  | RANGER | 0.996 (0.993–0.999) | 0.797 (0.723–0.871) | 0.983 | 0.590 |
|  | SVM | 0.882 (0.843–0.922) | 0.665 (0.558–0.772) | 0.810 | 0.456 |
|  | XGB | 0.907 (0.881–0.933) | 0.792 (0.719–0.864) | 0.722 | 0.556 |
|  | NB | 0.741 (0.690–0.793) | 0.787 (0.708–0.866) | 0.450 | 0.523 |
|  | NNET | 0.779 (0.733–0.825) | 0.781 (0.701–0.860) | 0.552 | 0.533 |

AUC, area under the receiver operating characteristic curve; AUPRC, area under the precision–recall curve; GBM, gradient boosting machine; GLM, generalized linear model (logistic regression); GLMNET, penalized logistic regression (elastic net); RANGER, random forest; SVM, support vector machine; XGB, extreme gradient boosting; NB, naïve Bayes; NNET, neural network.
